# Mental Health Among Adults with Diabetes in Spain: A Population-Based Matched Study of Emotional Well-Being, Depressive Symptoms, and Psychiatric Medication Use

**DOI:** 10.3390/healthcare14172691

**Published:** 2026-08-24

**Authors:** Ana López-de-Andrés, Tomás Chivato-Martin-Falquina, Rodrigo Jiménez-García, José J. Zamorano-León, David Carabantes-Alarcon, Andrés Bodas-Pinedo, Diana Maria Merida, Lucia Fuentes-Arroyo, Lucia Jiménez-Sierra

**Affiliations:** 1 Department of Public Health and Maternal & Child Health, Faculty of Pharmacy, Universidad Complutense de Madrid, Instituto de Investigación Sanitaria del Hospital Clínico San Carlos (IdISSC), 28040 Madrid, Spain; anailo04@ucm.es; 2 Unidad de Radiofarmacia, Hospital General Universitario Santa Lucía, 30202 Cartagena, Spain; tomas.chivato@carm.es; 3 Instituto de Investigación Sanitaria del Hospital Clínico San Carlos (IdISSC), 28040 Madrid, Spain; rodrijim@ucm.es (R.J.-G.); dcaraban@ucm.es (D.C.-A.); abodas@ucm.es (A.B.-P.); dmerida@ucm.es (D.M.M.); luciaifu@ucm.es (L.F.-A.); 4 Department of Public Health and Maternal & Child Health, Faculty of Medicine, Universidad Complutense de Madrid, 28040 Madrid, Spain; lujime05@ucm.es

**Keywords:** diabetes mellitus, mental health, emotional well-being, depressive symptoms, psychiatric medication use, social support, physical activity, sex differences, Spain

## Abstract

**Background/Objectives:** The objectives of this study were to compare emotional well-being, depressive symptoms, and psychiatric medication use between adults with and without diabetes in Spain and to identify sex-specific factors associated with these outcomes among individuals with diabetes. **Methods:** A population-based matched study was conducted using data from the 2023 Spanish National Health Survey (SNHS 2023). Adults aged ≥35 years with physician-diagnosed diabetes were individually matched (1:1) to controls without diabetes according to sex, exact age, and autonomous community of residence. Low emotional well-being (WHO-5 score < 50), clinically relevant depressive symptoms (PHQ-8 ≥ 10), and psychiatric medication use were assessed. Conditional logistic regression and sex-specific multivariable logistic regression models were fitted. **Results:** The study included 3710 participants (1855 with diabetes and 1855 matched controls without diabetes). The prevalence of low emotional well-being was significantly higher among participants with diabetes than among matched controls in both men (16.4% vs. 11.6%; *p* = 0.004) and women (28.8% vs. 21.6%; *p* < 0.001). Likewise, clinically relevant depressive symptoms were more frequent among individuals with diabetes (men: 32.7% vs. 24.9%, *p* < 0.001; women: 54.4% vs. 40.2%, *p* < 0.001), as was psychiatric medication use (men: 20.5% vs. 15.2%, *p* = 0.005; women: 39.5% vs. 30.2%, *p* < 0.001). After full adjustment, diabetes remained independently associated with low emotional well-being (men: aOR 1.48, 95% CI 1.03–2.13; women: aOR 1.43, 95% CI 1.14–1.78), depressive symptoms (men: aOR 1.31, 95% CI 1.02–1.69; women: aOR 1.38, 95% CI 1.08–1.77), and psychiatric medication use (men: aOR 1.34, 95% CI 1.02–1.75; women: aOR 1.35, 95% CI 1.06–1.73). Poor social support, physical inactivity, and several chronic comorbidities emerged as the factors most consistently associated with adverse mental health outcomes among individuals with diabetes. **Conclusions:** Adults with diabetes in Spain experience a substantially greater burden of adverse mental health than matched individuals without diabetes. Integrating routine mental health assessment and psychosocial support into diabetes care may help improve both psychological well-being and disease management.

## 1. Introduction

Diabetes mellitus is one of the most prevalent chronic diseases worldwide and a leading contributor to global morbidity and mortality. Recent estimates indicate that the number of adults living with diabetes has increased substantially over the last three decades and is expected to continue rising through 2050 [1,2]. Consequently, diabetes represents a major challenge for healthcare systems and public health worldwide [1].

Beyond its well-recognized physical complications, diabetes can also adversely affect mental health and psychological well-being [3]. Individuals living with diabetes must continuously manage treatment requirements, lifestyle modifications, glucose monitoring, and concerns regarding disease progression and complications, all of which may impose a considerable psychological burden and contribute to diabetes-related distress [4]. These challenges may negatively influence emotional well-being and increase vulnerability to mental health disorders. Furthermore, the relationship between diabetes and mental health appears to be bidirectional, as psychological disorders may impair self-care behaviors, treatment adherence, and glycemic control, while poor diabetes outcomes may in turn exacerbate psychological distress and mental health problems [5].

Among mental health conditions, depression has received particular attention in the literature of diabetes. Numerous epidemiological studies and meta-analyses have consistently shown that individuals with diabetes experience a substantially higher prevalence of depressive symptoms and depressive disorders than the general population [6,7]. Depression in people with diabetes has been associated with poorer glycemic control, impaired self-management, an increased risk of diabetes-related complications, and higher mortality rates [8,9,10]. However, mental health encompasses broader dimensions than the presence or absence of depressive disorders alone [11,12]. Mental health in people with diabetes encompasses not only depressive symptoms but also positive psychological functioning [13,14,15,16,17,18,19]. Accordingly, the simultaneous assessment of emotional well-being using the World Health Organization-Five Well-Being Index (WHO-5) and depressive symptoms using the eight-item Patient Health Questionnaire (PHQ-8) allows complementary dimensions of mental health to be evaluated [14,17,20]. The PHQ-8 is a validated measure of the presence and severity of depressive symptoms, whereas the WHO-5 assesses subjective psychological well-being through positively worded items covering positive mood, vitality, calmness, restfulness, and interest in daily life [14,17,20]. Although closely related, these constructs are not interchangeable. This distinction is consistent with the dual-continua model, which conceptualizes positive mental health and mental illness as correlated but distinct dimensions; therefore, the absence of clinically relevant depressive symptoms does not necessarily imply adequate emotional well-being [21]. More broadly, positive well-being reflects individuals’ capacity to function, adapt, cope with adversity, and participate in daily life, aspects that are not fully captured by symptom-based measures alone [22,23]. This distinction is particularly relevant in diabetes. Beyond depression, the continuous demands of self-management, including glucose monitoring, medication use, dietary decisions, fear of hypoglycemia, and concerns about long-term complications, may substantially impair emotional well-being even in the absence of clinically relevant depressive symptoms [4,16,24]. Previous studies have reported poorer psychological well-being among people with diabetes and highlighted the importance of psychological adjustment and coping resources in shaping mental health outcomes [4,15,16,24]. Consistent with these observations, the WHO-5 has been recommended as an initial assessment of emotional well-being and a first-step screening instrument for depression in adults with diabetes [25]. Assessing both emotional well-being and depressive symptoms may therefore provide a more comprehensive characterization of the psychological burden associated with diabetes, facilitate the identification of vulnerable individuals who might otherwise remain undetected, and better inform public health and clinical interventions aimed at improving both psychological and physical health outcomes [14,15,16,17,18,19,24,25].

Likewise, psychiatric medication may be considered an additional indicator of mental health burden and healthcare needs within the population [26,27].

Several sociodemographic, clinical, and lifestyle-related factors may influence mental health outcomes among people with diabetes. Previous studies have identified poor social support, physical inactivity, obesity, and chronic comorbidities as important factors associated with adverse psychological outcomes, with social support emerging as one of the most consistent protective factors [28,29]. In addition, accumulating evidence suggests that men and women may experience the psychological consequences of diabetes differently, potentially reflecting biological, behavioral, and social mechanisms [30,31]. However, findings regarding sex-specific patterns remain inconsistent and further research is needed.

Despite the growing recognition of the importance of mental health in diabetes care, several important gaps remain. Most population-based studies have focused primarily on depressive symptoms, whereas other relevant dimensions of mental health, such as emotional well-being and psychiatric medication use, have received considerably less attention [3,4,5,6,10,11,14,15,16,17,18,19,26,27]. Furthermore, relatively few studies have simultaneously examined these mental health indicators separately in men and women with diabetes using survey data.

The aim of this study was to investigate differences in mental health indicators between adults with physician-diagnosed diabetes and matched individuals without diabetes in Spain using data from the Spanish National Health Survey (SNHS 2023). Specifically, we compared the prevalence of low emotional well-being, clinically relevant depressive symptoms, and psychiatric medication use between adults with and without diabetes. We also identified sex-specific factors associated with these mental health outcomes among individuals with diabetes and evaluated whether diabetes was independently associated with adverse mental health outcomes after controlling for sociodemographic characteristics, chronic health conditions, perceived social support, and lifestyle-related factors.

## 2. Materials and Methods

### 2.1. Study Design and Data Source

This study was based on data from the SNHS 2023, a nationally representative survey conducted by the Spanish Ministry of Health (SMH) in collaboration with the National Statistics Institute (Instituto Nacional de Estadística). The SNHS 2023 collects information on health status, chronic conditions, health-related behaviors, and sociodemographic characteristics among the non-institutionalized population residing in Spain [32].

The survey employed a stratified multistage sampling design to ensure representativeness at both national and regional levels. Census tracts were selected as primary sampling units, households were selected as secondary units, and one eligible adult per household was randomly selected for participation. Data collection was carried out between September 2023 and August 2024 using a mixed-mode methodology that combined interviewer-administered questionnaires and secure web-based data collection. Further details regarding the survey methodology are available elsewhere [32].

### 2.2. Study Population and Matching Procedure

The study population was restricted to adults aged 35 years or older who participated in the SNHS 2023. Diabetes status was determined based on self-reported physician-diagnosed diabetes. The SNHS 2023 does not differentiate between type 1 and type 2 diabetes; therefore, all adults reporting a physician diagnosis of diabetes were included. To minimize potential inclusion of type 1 diabetes, analyses were restricted to participants aged ≥35 years, among whom most diabetes cases are expected to have type 2 diabetes [33]. Participants reporting a previous physician diagnosis of diabetes were classified as having diabetes, whereas all remaining participants were considered eligible controls.

To improve comparability between groups and reduce potential confounding, a matched analytical design was implemented. Participants with diabetes were individually matched in a 1:1 ratio to participants without diabetes according to sex, exact age, and autonomous community of residence using exact matching procedures. Matching strata were generated from all possible combinations of these variables. When more than one eligible control was available within a given matching stratum, controls were randomly selected using a computer-generated randomization procedure. Matching was performed without replacement, ensuring that each control participant could be included only once in the final analytical dataset.

### 2.3. Main Outcome Variables

Three mental health indicators were considered as study outcomes: low emotional well-being, clinically relevant depressive symptoms, and psychiatric medication use.

Emotional well-being was assessed using WHO-5, a validated instrument composed of five positively worded items evaluating psychological well-being during the previous two weeks. Raw scores were transformed to a standardized scale ranging from 0 to 100, with higher values indicating better well-being. Consistent with previous studies, a WHO-5 score below 50 was considered indicative of low emotional well-being [20,34].

Clinically relevant depressive symptoms were evaluated using the PHQ-8, which assesses the frequency of depressive symptoms experienced during the previous two weeks. Total scores range from 0 to 24, with higher scores indicating greater symptom severity. In accordance with validated cut-off points, a PHQ-8 ≥ 10 was considered indicative of clinically relevant depressive symptoms [13].

Psychiatric medication use was assessed according to self-reported consumption of medications prescribed for mental health conditions during the previous two weeks. Participants were classified as psychiatric medication users when they reported the use of “antidepressants” and/or “tranquilizers, sedatives or sleeping medications”. In the SNHS 2023, the latter three medication types are collected in a single survey question and therefore cannot be analyzed separately.

### 2.4. Independent Study Variables

The main exposure variable was self-reported physician-diagnosed diabetes (yes/no).

Additional covariates included sociodemographic characteristics, perceived social support, health-related variables, and lifestyle factors. Sociodemographic variables comprised age group (35–59, 60–69, 70–79, and ≥80 years), educational attainment, employment status, monthly household income, and living with a partner.

Perceived social support was assessed using the Oslo Social Support Scale (OSSS-3) and categorized as poor, moderate, or strong social support according to established recommendations [35].

Health-related variables included self-rated health and selected physician-diagnosed chronic conditions. Self-rated health was categorized as very good/good or fair/bad/very bad. Chronic conditions considered in the analysis were cardiovascular diseases (including heart disease and stroke), musculoskeletal disorders, respiratory diseases, gastrointestinal disorders, neurological diseases, malignant tumors and hypertension. The number of chronic conditions was calculated by adding the seven possible chronic conditions collected for each participant.

Lifestyle-related variables included smoking status, alcohol consumption, leisure-time physical activity, and body mass index (BMI). Smoking status was categorized as current smoker, former smoker, or never smoker. Alcohol consumption was classified as daily or weekly consumption, occasional consumption (a few times per year), and no alcohol consumption during the previous year or lifetime abstention. Leisure-time physical activity was categorized as physically active or inactive according to self-reported practice of physical activity during leisure time. BMI was calculated from self-reported weight and height and categorized as normal weight (<25 kg/m^2^), overweight (25.0–29.9 kg/m^2^), or obesity (≥30 kg/m^2^).

Detailed definitions and categorizations of all variables included in the analyses are provided in Table S1.

### 2.5. Statistical Analysis

Descriptive analyses were used to summarize the sociodemographic, health-related, lifestyle, and mental health characteristics of participants with diabetes and matched controls without diabetes. Categorical variables were expressed as absolute frequencies and percentages.

Continuous variables were summarized as means and standard deviations (SD). Differences in continuous variables between participants with diabetes and matched controls without diabetes were assessed using paired Student’s *t*-tests. For continuous variables included in subgroup analyses according to mental health outcomes, mean values were compared between participants with diabetes and matched controls using linear regression models with cluster-robust standard errors to account for the matched-pair design.

The prevalence of low emotional well-being, clinically relevant depressive symptoms, and psychiatric medication use was estimated according to sociodemographic, health-related, and lifestyle characteristics among participants with and without diabetes. To compare participants with diabetes and matched controls without diabetes within each category of the study variables, separate conditional logistic regression models were fitted, accounting for the matched-pair design.

To assess factors associated with each mental health outcome separately among participants with and without diabetes, sex-specific bivariate logistic regression analyses were conducted. For categorical variables with more than two categories, overall statistical significance was evaluated using Wald tests.

Factors independently associated with low emotional well-being, clinically relevant depressive symptoms, and psychiatric medication use among participants with diabetes were identified through separate sex-specific multivariable logistic regression models. Variables considered clinically relevant and those associated with the outcome in bivariate analyses were evaluated for inclusion in the multivariable models. Model building followed the recommendations proposed by Hosmer et al. [36]. Non-significant variables were sequentially removed while assessing changes in regression coefficients to identify potential confounding effects. Adjusted odds ratios (aORs) and their corresponding 95% confidence intervals (95% CIs) were calculated.

Finally, the independent association between diabetes and each mental health outcome was examined using sex-stratified conditional logistic regression models. Two models were constructed for each outcome. Model 1 estimated the unadjusted association between diabetes and the outcome of interest. Model 2 was additionally adjusted for sociodemographic characteristics, perceived social support, chronic health conditions, and lifestyle-related factors. Results were expressed as odds ratios (ORs) for Model 1 and adjusted odds ratios (aORs) for Model 2, with corresponding 95% confidence intervals (95% CIs), using participants without diabetes as the reference group.

All regression analyses were performed using the matched analytical dataset to preserve the matched-pair structure. Statistical significance was established at a two-sided *p*-value < 0.05. Analyses were conducted using Stata 15.1 statistical software (StataCorp, College Station, TX, USA).

### 2.6. Sensitivity Analysis

To assess the potential impact of missing data, we compared participants with diabetes excluded because of missing information on any mental health outcomes with those with all these variables recorded and included before the matching procedure.

### 2.7. Ethical Considerations

All participants provided informed consent for participation in the SNHS 2023 in accordance with the procedures established for the national survey [32]. The present study involved a secondary analysis of anonymized, publicly available SNHS data and did not involve direct contact with participants; therefore, no additional informed consent was required for the present analysis. As the present study was based on a secondary analysis of anonymized public-use data, additional ethical approval was not required according to Spanish legislation [37,38].

## 3. Results

**Figure 1:**
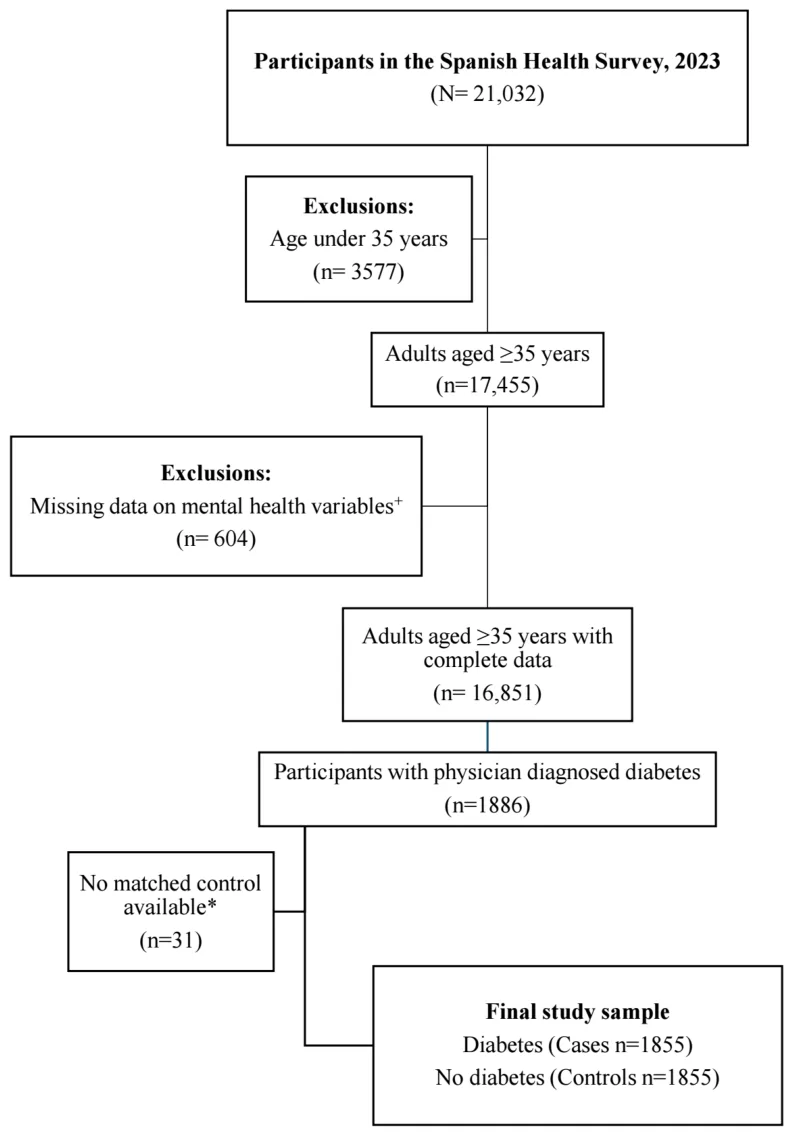
Flowchart of participant selection and matching process. * Each participant with diabetes (case) was individually matched 1:1 to a participant without diabetes (control) by exact age, sex, and autonomous community of residence. ^+^ WHO-5, World Health Organization-Five Well-Being Index. PHQ-8, Patient Health Questionnaire eight-item. Psychiatric medication use during the last two weeks was assessed.

A total of 21,032 individuals participated in the SNHS 2023. After excluding 3577 participants aged under 35 years, 17,455 adults aged ≥35 years remained eligible for the study. A further 604 individuals were excluded because of missing information on the mental health variables of interest, resulting in 16,851 adults with complete data. The flowchart for participant selection is shown in Figure 1. Among eligible participants, 1855 adults with physician-diagnosed diabetes could be exactly matched to a control without diabetes according to sex, exact age, and autonomous community of residence. Consequently, the final study population comprised 3710 participants, including 1855 adults with diabetes and 1855 matched controls without diabetes.

The mean age of the study population was 70.4 years (SD 12.2). Overall, 1950 participants (52.6%) were women and 1760 (47.4%) were men.

### 3.1. Characteristics of the Study Population

The sociodemographic, health-related, lifestyle, and mental health characteristics of the study population are presented in Table 1 for men and Table 2 for women.

Among men, participants with diabetes and matched controls were comparable regarding educational level, employment status, living with a partner, and perceived social support. However, men with diabetes reported significantly poorer self-rated health and higher prevalences of cardiovascular disease (30.8% vs. 18.0%; *p* < 0.001), hypertension (62.5% vs. 42.8%; *p* < 0.001), musculoskeletal disorders (46.6% vs. 33.3%; *p* < 0.001), and gastrointestinal disease (22.3% vs. 17.0%; *p* = 0.005). The mean number of chronic conditions was significantly higher (1.89 vs. 1.34; *p* < 0.001). They were also less likely to be physically active (13.6% vs. 20.2%; *p* < 0.001), consumed alcohol less frequently, and showed a higher prevalence of obesity (29.0% vs. 18.9%; *p* < 0.001). Furthermore, low emotional well-being (16.4% vs. 11.6%; *p* = 0.004), depressive symptoms (32.7% vs. 24.9%; *p* < 0.001), and psychiatric medication use (20.5% vs. 15.2%; *p* = 0.005) were significantly more common among men with diabetes (Table 1).

As shown in Table 2, women with diabetes had lower educational attainment and lower employment rates than their matched controls. They also reported poorer self-rated health and a significantly higher prevalence of all chronic conditions analyzed, including cardiovascular disease, hypertension, musculoskeletal disorders, respiratory disease, gastrointestinal disease, neurological disease, and malignant tumors. The mean number of chronic conditions was 2.32 among women with diabetes and 1.67 among matched controls (*p* < 0.001). Women with diabetes were less physically active, reported lower alcohol consumption, and had almost twice the prevalence of obesity compared with women without diabetes (30.5% vs. 15.9%; *p* < 0.001). All mental health outcomes were significantly more frequent among women with diabetes, including low emotional well-being (28.8% vs. 21.6%; *p* < 0.001), depressive symptoms (54.4% vs. 40.2%; *p* < 0.001), and psychiatric medication use (39.5% vs. 30.2%; *p* < 0.001).

Table 3 and Table 4 present the prevalence of low emotional well-being, clinically relevant depressive symptoms, and psychiatric medication use according to sociodemographic, health-related, and lifestyle characteristics among men with diabetes and matched controls without diabetes.

Compared with matched controls, men with diabetes consistently showed a higher prevalence of low emotional well-being across most study categories. Significant differences were observed according to age group, with the highest prevalence among men aged ≥80 years (23.7% vs. 15.8%; *p* = 0.015). Higher prevalences were also found among those living with a partner (15.9% vs. 10.3%; *p* = 0.011), ex-smokers (16.5% vs. 11.0%; *p* = 0.018), participants with obesity (16.1% vs. 11.2%; *p* = 0.024), and those without respiratory, gastrointestinal, or neurological diseases (all *p* < 0.01). Among men with diabetes, low emotional well-being was significantly associated with age group, social support, self-rated health, cardiovascular disease, musculoskeletal disorders, respiratory disease, gastrointestinal disease, neurological disease, and hypertension (Table 3 and Table 4).

The prevalence of clinically relevant depressive symptoms was also significantly higher among men with diabetes than among matched controls in several categories. The largest differences were observed among participants aged ≥80 years (46.9% vs. 32.6%; *p* = 0.004), retired or disabled individuals (34.7% vs. 25.7%; *p* < 0.001), those reporting moderate or strong social support (both *p* < 0.001), physically inactive men (34.8% vs. 27.3%; *p* < 0.001), participants with overweight (30.1% vs. 23.0%; *p* = 0.003), and those who reported no alcohol consumption during the previous year or lifetime abstention (42.0% vs. 34.4%; *p* = 0.005). Within the diabetes group, clinically relevant depressive symptoms were significantly associated with age group, employment status, social support, self-rated health, cardiovascular disease, musculoskeletal disorders, respiratory disease, gastrointestinal disease, neurological disease, alcohol consumption, and physical inactivity.

Psychiatric medication use was generally more common among men with diabetes than among controls. Significant differences were identified according to age group, particularly among men aged 60–69 years (16.5% vs. 14.1%; *p* = 0.018) and 70–79 years (19.6% vs. 16.3%; *p* < 0.001). Higher prevalences were also observed among participants living with a partner (19.2% vs. 13.4%; *p* = 0.006), ex-smokers (22.3% vs. 17.1%; *p* = 0.006), individuals with overweight (17.9% vs. 14.5%; *p* = 0.041), those with gastrointestinal disease (34.7% vs. 21.3%; *p* = 0.032), and those who reported no alcohol consumption during the previous year or lifetime abstention (25.7% vs. 17.7%; *p* = 0.039). Among men with diabetes, psychiatric medication use was significantly associated with age group, employment status, self-rated health, cardiovascular disease, musculoskeletal disorders, respiratory diseases, gastrointestinal diseases, neurological diseases and alcohol consumption.

Among men with diabetes, the mean values for the number of chronic conditions were significantly higher than among controls without diabetes for the three mental health outcomes studied.

### 3.2. Mental Health Outcomes Among Women According to Study Variables

The prevalence of mental health outcomes among women according to sociodemographic, health-related, and lifestyle characteristics is shown in Table 5 and Table 6.

Compared with matched controls, women with diabetes consistently showed a higher prevalence of low emotional well-being across most study categories. Significant differences were observed according to age group, with the highest prevalence among women aged ≥80 years (40.5% vs. 33.3%; *p* = 0.015). Higher prevalences were also found among women living without a partner (31.0% vs. 24.6%; *p* = 0.033), those reporting poor social support (62.7% vs. 32.7%; *p* = 0.001), never smokers (28.8% vs. 23.3%; *p* = 0.024), women with obesity (34.9% vs. 25.9%; *p* = 0.024), physically inactive women (30.5% vs. 23.2%; *p* < 0.001), and those without gastrointestinal or neurological diseases (both *p* < 0.01). Within the diabetes group, low emotional well-being was significantly associated with age group, educational attainment, employment status, social support, self-rated health, cardiovascular disease, musculoskeletal disorders, respiratory disease, gastrointestinal disease, neurological disease, hypertension, malignant tumors, alcohol consumption, physical inactivity, and body weight status.

The prevalence of clinically relevant depressive symptoms was markedly higher among women with diabetes than among matched controls in most categories. The largest significant differences were observed among women aged 35–59 years (49.0% vs. 28.0%; *p* < 0.001), 60–69 years (46.2% vs. 28.1%; *p* = 0.001), and ≥80 years (66.3% vs. 59.5%; *p* = 0.004), as well as among retired or disabled women (56.7% vs. 43.7%; *p* < 0.001), those living without a partner (58.2% vs. 43.2%; *p* < 0.001), women reporting moderate or strong social support (both *p* < 0.001), physically inactive women (56.7% vs. 43.0%; *p* < 0.001), women with overweight (51.5% vs. 40.4%; *p* = 0.003), and those who had not consumed alcohol during the previous year or were lifetime abstainers (57.3% vs. 48.3%; *p* = 0.005). Among women with diabetes, clinically relevant depressive symptoms were significantly associated with age group, living with a partner, social support, self-rated health, cardiovascular disease, musculoskeletal disorders, respiratory disease, gastrointestinal disease, neurological disease, malignant tumors, alcohol consumption, and physical inactivity.

Psychiatric medication use was also generally more prevalent among women with diabetes than among controls. Significant differences were identified among women aged 35–59 years (27.0% vs. 16.0%; *p* = 0.016), 60–69 years (33.9% vs. 22.0%; *p* = 0.018), and 70–79 years (41.3% vs. 26.2%; *p* < 0.001), as well as among retired or disabled women (45.0% vs. 34.1%; *p* = 0.001), women living without a partner (41.8% vs. 32.4%; *p* = 0.026), those reporting poor social support (46.3% vs. 22.4%; *p* = 0.008) and moderate social support (43.4% vs. 26.7%; *p* = 0.001), physically inactive women (39.8% vs. 31.7%; *p* < 0.001), never smokers (39.0% vs. 31.8%; *p* = 0.003), women with overweight (39.4% vs. 28.8%; *p* = 0.041), and women with gastrointestinal disease (54.7% vs. 46.8%; *p* = 0.032). Within the diabetes group, psychiatric medication use was significantly associated with age group, educational attainment, employment status, social support, self-rated health, cardiovascular disease, hypertension, musculoskeletal disorders, gastrointestinal disease, neurological disease, and malignant tumors. As observed among men, women with diabetes had a higher mean number of chronic conditions than women without diabetes across all three mental health outcomes analyzed.

### 3.3. Factors Independently Associated with Mental Health Outcomes Among Participants with Diabetes

Figures S1–S6 present the multivariable unconditional logistic regression models identifying factors independently associated with low emotional well-being, clinically relevant depressive symptoms, and psychiatric medication use among participants with diabetes.

Among men with diabetes, poor social support emerged as the strongest association for both low emotional well-being (aOR = 3.80; 95% CI: 1.82–7.92) and depressive symptoms (aOR = 5.32; 95% CI: 2.57–10.99). Neurological disorders were independently associated with all three mental health outcomes, including low emotional well-being (aOR = 2.51; 95% CI: 1.34–4.70), depressive symptoms (aOR = 3.29; 95% CI: 1.69–6.40), and psychiatric medication use (aOR = 2.48; 95% CI: 1.38–4.45). Gastrointestinal disorders also remained independently associated with adverse mental health outcomes, whereas leisure-time physical activity was associated with a lower likelihood of depressive symptoms (aOR = 0.42; 95% CI: 0.24–0.74).

Among women with diabetes, poor social support was likewise the strongest factor associated with adverse mental health outcomes, increasing the odds of low emotional well-being almost tenfold (aOR = 9.49; 95% CI: 4.74–18.98) and depressive symptoms more than fivefold (aOR = 5.42; 95% CI: 2.68–10.98). Tumors, cardiovascular disease, musculoskeletal disorders, and gastrointestinal disease remained independently associated with adverse mental health outcomes. Leisure-time physical activity showed a strong association with both low emotional well-being (aOR = 0.27; 95% CI: 0.11–0.67) and depressive symptoms (aOR = 0.41; 95% CI: 0.24–0.69), while obesity remained independently associated with low emotional well-being (aOR = 1.68; 95% CI: 1.00–2.82). Neurological disorders were strongly associated with psychiatric medication use (aOR = 2.49; 95% CI: 1.66–3.74).

### 3.4. Association Between Diabetes and Mental Health Outcomes: Multivariable Analysis

The association between diabetes and mental health outcomes after multivariable conditional logistic regression is shown in Table 7.

In crude analyses, diabetes was significantly associated with all adverse mental health outcomes in both sexes. Among men, diabetes was associated with increased odds of low emotional well-being (OR = 1.51; 95% CI: 1.14–2.00), depressive symptoms (OR = 1.52; 95% CI: 1.22–1.90), and psychiatric medication use (OR = 1.42; 95% CI: 1.11–1.82). Similar associations were observed among women, with crude ORs ranging from 1.48 for low emotional well-being to 1.93 for depressive symptoms.

After adjustment for sociodemographic characteristics, social support, chronic conditions, and lifestyle-related factors, diabetes remained independently associated with all mental health outcomes. Among men, diabetes was associated with low emotional well-being (aOR = 1.48; 95% CI: 1.03–2.13), depressive symptoms (aOR = 1.31; 95% CI: 1.02–1.69), and psychiatric medication use (aOR = 1.34; 95% CI: 1.02–1.75). Likewise, women with diabetes had higher odds of low emotional well-being (aOR = 1.43; 95% CI: 1.14–1.78), depressive symptoms (aOR = 1.38; 95% CI: 1.08–1.77), and psychiatric medication use (aOR = 1.35; 95% CI: 1.06–1.73) than their matched counterparts without diabetes. Although the magnitude of the associations decreased after adjustment, diabetes remained independently associated with adverse mental health outcomes in both men and women.

Table S2 compares the characteristics of participants with diabetes who were excluded because of missing information on mental health outcomes (n = 86) with those who had complete outcome data before matching. Compared with those participants with complete information, excluded participants were generally older and had poorer self-rated health, lower perceived social support, and a higher prevalence of neurological disorders, whereas no major differences were observed regarding sex, educational level, employment status, household income, or most lifestyle-related characteristics. When we compared the mental health outcomes recorded among those with at least one missing variable, and excluded, with those with all data recorded, no significant differences were found.

## 4. Discussion

In this matched study based on data from a national population-based survey of Spanish adults aged 35 years and older, individuals with diabetes exhibited a significantly higher prevalence of low emotional well-being, clinically relevant depressive symptoms, and psychiatric medication use than matched controls without diabetes. These associations remained statistically significant after adjustment for sociodemographic characteristics, perceived social support, chronic health conditions, and lifestyle-related factors, suggesting that diabetes was independently associated with adverse mental health outcomes. In addition, several factors were independently associated with poor mental health among participants with diabetes, particularly poor social support, physical inactivity, obesity, and selected chronic comorbidities. Notably, the magnitude and pattern of these associations differed between men and women, underscoring the importance of considering sex-specific variables when addressing mental health in people living with diabetes.

Our findings regarding depressive symptoms are consistent with a large body of epidemiological evidence showing that individuals with diabetes are more likely to experience depression than those without the disease. In the present study, diabetes was associated with a significantly higher prevalence of clinically relevant depressive symptoms in both men and women. These findings are in line with previous systematic reviews and meta-analyses reporting a substantially greater burden of depression among people living with diabetes compared with the general population [5,6,7,10,14].

Our results are also consistent with previous evidence from Spain. Using a population-based matched case–control design, López-Herranz et al. reported significantly poorer mental health among Spanish adults with diabetes compared with matched controls, supporting the existence of a persistent association between diabetes and adverse psychological outcomes in this population [39]. The consistency between that study and our findings is particularly noteworthy given the different survey periods and the substantial social and healthcare changes that have occurred in recent years.

Several biological and psychosocial mechanisms have been proposed to explain the association between diabetes and poor mental health [3,5]. Proposed biological pathways linking diabetes and depression include hypothalamic–pituitary–adrenal axis dysregulation, inflammatory processes, and hyperglycemia-related metabolic and vascular changes, although their causal contributions remain incompletely understood [3,5,40]. Psychosocial mechanisms like the daily demands associated with diabetes self-management, including glucose monitoring, dietary restrictions, medication adherence, treatment burden, and concerns regarding future complications, may generate chronic psychological stress and increase vulnerability to depression [3,5,41]. Conversely, depression is associated with poorer adherence to medication, diet, physical activity, glucose monitoring, and other self-care behaviors, which may contribute to poorer glycemic control [3,5,10,40,41]. These findings are particularly relevant because depression among individuals with diabetes has been associated with an increased risk of diabetes-related complications, poorer disease management, and higher mortality [3,5,8,10,40,41,42]. Taken together, the available evidence and our findings support the need for routine assessment of depressive symptoms as part of comprehensive diabetes care and population-based strategies aimed at improving the overall health of people living with diabetes.

An important finding of the present study was the higher prevalence of low emotional well-being among adults with diabetes compared with matched controls. Although the association between diabetes and depression has been extensively investigated, considerably less attention has been paid to positive dimensions of mental health, such as emotional well-being and psychological functioning [15,16,19,24]. Consequently, our findings contribute to a growing body of evidence suggesting that the mental health burden associated with diabetes extends beyond the presence of depressive symptoms alone.

Consistent with previous studies using the WHO-5, the lower emotional well-being observed among adults with diabetes may reflect the considerable psychological and behavioral burden associated with living with the disease [43,44,45,46]. Daily self-management, including blood glucose monitoring, medication adherence, dietary restrictions, and regular physical activity, requires sustained effort and may contribute to chronic stress and anxiety, ultimately reducing positive well-being [43,44,45,46]. In addition, concerns about the development of long-term complications and fear of hypoglycemia, particularly among individuals receiving insulin therapy, have been consistently associated with lower WHO-5 scores and poorer health-related quality of life [5,43,44,45,46]. Adults with diabetes are also more likely to experience comorbid conditions, such as obesity, hypertension, and cardiovascular disease, which increase physical limitations and negatively affect emotional well-being. Sleep disturbances, fatigue, frequent healthcare utilization, and socioeconomic challenges may further contribute to the lower WHO-5 scores observed in this population. Overall, evidence from studies using the WHO-5 suggests that reduced emotional well-being in adults with diabetes reflects the cumulative impact of diabetes-related anxiety, the ongoing demands of self-management, comorbidity burden, and broader biopsychosocial factors, rather than the disease itself in isolation [5,43,44,45,46].

From an epidemiological perspective, our findings support the notion that assessing mental health exclusively through depressive symptoms may underestimate the broader psychological impact of diabetes. Individuals with diabetes may experience reduced emotional well-being without fulfilling diagnostic criteria for depression, highlighting the value of incorporating measures of positive mental health into population-based surveys and routine clinical assessments [17].

Another relevant finding of our study was the higher prevalence of psychiatric medication use among adults with diabetes compared with matched controls without diabetes. Although psychiatric medication use does not constitute a direct measure of mental health status, it may be considered a useful indicator of psychological burden, healthcare needs, and contact with mental health services at the population level.

Our findings are consistent with previous evidence from Spain. López-Herranz et al. reported a significantly higher prevalence of psychotropic medication use among Spanish adults with diabetes than among matched controls, supporting the notion that mental health-related healthcare needs are greater in this population [39]. Similar observations have been reported in other settings, where people with diabetes were more likely to require psychological support and mental healthcare services than individuals without diabetes [18].

The higher use of psychiatric medications observed among participants with diabetes is likely multifactorial. The coexistence of depressive symptoms, reduced emotional well-being, diabetes-related distress, and multiple chronic comorbidities may increase both the recognition of psychological problems and the likelihood of receiving pharmacological treatment [5,19]. Furthermore, previous studies suggest that individuals with diabetes receiving psychotropic medications often represent a particularly vulnerable subgroup characterized by a higher burden of physical and psychological morbidity [27,47]. From a public health perspective, these findings reinforce the importance of integrating mental health assessment into routine diabetes care, as a substantial proportion of people living with diabetes appear to experience mental health needs that may require clinical attention.

Several factors were independently associated with adverse mental health outcomes among participants with diabetes, particularly poor social support, physical inactivity, obesity, and the presence of chronic comorbidities. Among these, perceived social support emerged as one of the strongest and most consistent correlates of mental health across all outcomes and in both sexes. Individuals reporting poor social support showed substantially higher odds of low emotional well-being, depressive symptoms, and psychiatric medication use, with especially pronounced associations among women. These findings are consistent with previous evidence indicating that inadequate social support is associated with psychological vulnerability in people with diabetes. A recent systematic review and meta-analysis reported that individuals with type 2 diabetes and low social support had approximately twice the risk of depression compared with those receiving adequate support [28]. Social support may exert its beneficial effects through multiple pathways, including improved coping abilities, enhanced self-efficacy, reduced diabetes-related distress, and better adherence to self-management recommendations [48].

Previous research suggests that household income may be associated with mental health in people with diabetes, with lower household-income categories being associated with poorer self-rated general health and greater reported mental distress among US adults with diabetes [49]. However, in our study, household income was not associated with mental health outcomes in bivariate or after multivariable adjustment, suggesting that this association may be explained by other social factors. Physical activity was also associated with more favorable mental health profiles. In our study, physically active participants with diabetes generally exhibited more favorable mental health profiles than inactive individuals. This observation is consistent with robust epidemiological evidence demonstrating an inverse association between physical activity and depression. A large meta-analysis including almost two million participants found that even moderate levels of physical activity were associated with a significantly lower risk of depression [29]. Furthermore, evidence specific to diabetes suggests that exercise interventions can effectively reduce depressive symptoms and improve psychological well-being among individuals living with the disease [50].

Obesity and chronic comorbidities were also associated with poorer mental health outcomes. The relationship between obesity and adverse mental health appears to be bidirectional, with obesity increasing the risk of depression and depression contributing to weight gain over time [51]. In addition, recent studies among individuals with diabetes have shown that obesity frequently coexists with higher levels of diabetes-related distress and poorer psychological functioning [52]. Similarly, the presence of multiple chronic conditions may increase treatment complexity, functional limitations, symptom burden, and concerns regarding future health, thereby negatively affecting mental health. Recent evidence suggests that multimorbidity contributes substantially to diabetes-related distress and diminished quality of life, reinforcing the importance of adopting a holistic approach when addressing the mental health needs of people living with diabetes [53].

An additional finding of our study was the existence of marked sex differences across all mental health indicators. Women with diabetes consistently exhibited a higher prevalence of low emotional well-being, clinically relevant depressive symptoms, and psychiatric medication use than men with diabetes. Furthermore, the association of some variables, particularly perceived social support, appeared to be stronger among women, among whom the largest odds ratios for adverse mental health outcomes were observed.

These findings are consistent with recent evidence from Spain. Using nationwide hospital data, Jiménez-Sierra et al. reported a significantly higher prevalence of depression among women with type 2 diabetes than among men, confirming the existence of persistent sex disparities in mental health outcomes in the Spanish diabetic population [54]. The stronger associations observed among women are consistent with previous international evidence showing that depression is more prevalent and psychological well-being is generally poorer in women with diabetes than in men [29,30,31,55,56]. Although differences in prevalence do not necessarily demonstrate that diabetes has a stronger psychological association in women, population-based evidence suggests that the association between diabetes and major depressive disorder may be more pronounced in women than in men [29,30,31,55,56]. Several biological and psychosocial mechanisms may contribute to these differences. Sex-related biological pathways, including hormonal influences and differences in inflammatory responses, may affect vulnerability to depression [57]. Gender-related social factors, including socioeconomic disadvantage and greater caregiving responsibilities, may further increase the burden of managing diabetes among women [29,30,31,55,56]. In addition, diabetes-related distress has been associated with female sex, while negative coping strategies appear to be more frequent among women and are associated with poorer quality of life [43]. It has been suggested that women with diabetes have more diabetes-related distress than men in relation to interpersonal relationships, family responsibilities, and social roles [58]. In addition, women generally present a higher burden of chronic conditions and may perceive a greater impact of diabetes on daily life and well-being [59]. Together, these factors may partly explain why women in our study consistently showed poorer outcomes across the different mental health indicators.

Of particular interest, poor social support showed especially strong associations with adverse mental health outcomes among women in our study. This finding suggests that psychosocial factors may be more strongly associated with mental health among women and could partly explain the higher prevalence of emotional and depressive symptoms observed in this group. The higher prevalence of psychiatric medication use among women is also consistent with evidence from Spain showing greater psychotropic medication prescribing among females than males in routine clinical practice [60].

Taken together, these findings highlight the need for sex-sensitive approaches to diabetes management and mental health promotion, recognizing that women may experience both a greater burden of psychological problems and stronger associations between adverse psychosocial factors and mental health outcomes.

This study has several strengths that should be acknowledged. First, it was based on data from the 2023 Spanish National Health Survey, a national survey of the adult population. Second, the matched design reduced the potential influence of important demographic confounders by ensuring comparability between participants with and without diabetes according to sex, exact age, and autonomous community of residence. Third, mental health was assessed using complementary indicators, including emotional well-being, clinically relevant depressive symptoms, and psychiatric medication use, allowing a broader evaluation of mental health than studies focusing exclusively on depression. Finally, the sex-stratified analyses enabled the identification of differential patterns and variables associated with mental health outcomes among men and women with diabetes.

Several limitations should also be considered. The cross-sectional design precludes establishing causal relationships between diabetes and the mental health outcomes evaluated. In addition, all information was based on self-report, which may be subject to recall and reporting biases. The SNHS does not distinguish between type 1 and type 2 diabetes. Diabetes prevalence increases markedly with age in Spain, and over 95% of diabetes cases in the Spanish population are estimated to be type 2 [33]. Therefore, because our analyses were restricted to adults aged ≥35 years, the findings are expected to primarily reflect type 2 diabetes, although some individuals with type 1 or other types of diabetes may have been included. An additional limitation is that diabetes status was based on self-reported physician-diagnosed diabetes. Although self-reported diabetes has shown good validity in Spanish population-based health surveys, its sensitivity is imperfect, and some misclassification—particularly the omission of undiagnosed cases—cannot be excluded [61,62]. In addition, the survey does not provide information on diabetes duration, glycemic control, diabetes-related complications, or treatment. These clinical characteristics are associated with psychosocial well-being among people with diabetes, and their absence prevented us from characterizing diabetes severity or examining whether the observed associations varied across clinical profiles [63]. Another limitation concerns the assessment of psychiatric medication use. In the Spanish National Health Survey, participants report physician-prescribed medications taken during the previous two weeks, including antidepressants and a single category comprising tranquilizers, sedatives or sleeping pills. Because these medications are not recorded separately and their clinical indication is unavailable, it was not possible to distinguish medications prescribed for depression or anxiety from those used primarily for sleep disturbances, neuropathic pain, or other clinical conditions. Moreover, psychotropic or antidepressant medication use has been documented among individuals without a recent or recorded psychiatric diagnosis [64,65]. Consequently, psychiatric medication use should be interpreted as a broad indicator of prescribed psychotropic treatment rather than as a direct measure of a specific psychiatric disorder. This distinction is particularly relevant given the high prevalence of sleep disturbances among people with type 2 diabetes; Riahi et al., for example, reported poor sleep quality in 70.3% of patients attending an Iranian diabetes clinic. Therefore, this outcome should be interpreted with care [66]. Although the study sample was derived from the SNHS 2023, a national population-based survey, survey weights could not be applied after the matching procedure. Therefore, the estimates obtained from the matched analytical sample should not be interpreted as nationally representative prevalence estimates, and their generalizability to the entire Spanish adult population should be considered with caution. Although participants were individually matched by exact age, sex, and autonomous community of residence, matching and multivariable adjustment cannot eliminate all confounding, particularly that arising from unmeasured or imperfectly measured factors [67,68]. Socioeconomic characteristics, obesity, comorbidities, and lifestyle factors differed between participants with and without diabetes and may have influenced the observed associations. Variables considered potential confounders on substantive grounds were therefore included as covariates in the multivariable regression models. Matching was limited to exact age, sex, and autonomous community because imposing additional matching criteria could have reduced statistical efficiency and restricted the number of participants for whom suitable matches could be identified [67]. Moreover, the inclusion of each additional variable in the matching or adjustment strategy requires careful consideration, because adjusting for variables affected by diabetes, including potential mediators, may result in overadjustment or selection bias [68]. Therefore, despite extensive covariate adjustment, unmeasured or residual confounding cannot be entirely ruled out.

The participation rate in the SNHS 2023 was 62.4%; therefore, the possibility of non-response bias should be considered [69]. Given that our study was restricted to adults aged ≥35 years, our findings are therefore expected to primarily reflect the mental health burden associated with type 2 diabetes. Finally, the study used a complete-case approach, excluding participants with missing information on any of the mental health outcomes analyzed. Compared with included participants, excluded individuals were older, had poorer self-rated health, lower perceived social support, and a higher prevalence of neurological disorders, while remaining broadly comparable regarding most sociodemographic characteristics, lifestyle factors and those mental health variables that could be compared. Although this provides some reassurance, it does not rule out selection bias. Therefore, the potential influence of missing outcome data on both prevalence estimates and adjusted associations should be acknowledged.

## 5. Conclusions

In conclusion, adults with diabetes in Spain exhibited significantly poorer mental health than matched individuals without diabetes, including lower emotional well-being, a higher prevalence of clinically relevant depressive symptoms, and greater use of psychiatric medications. These associations persisted after adjustment for sociodemographic characteristics, social support, chronic conditions, and lifestyle-related factors; diabetes remained independently associated with adverse mental health outcomes.

Poor social support, physical inactivity, and chronic comorbidities emerged as important factors associated with mental health among people with diabetes, while substantial sex differences were observed across all outcomes. These findings highlight the need to incorporate routine mental health assessment into diabetes care and to develop prevention and intervention strategies that address both psychosocial and lifestyle-related factors associated with mental health, with particular attention to vulnerable population groups.

## Figures and Tables

**Table 1:** Sociodemographic, health-related, lifestyle and mental health characteristics among men reporting diabetes and matched controls without diabetes in Spain (SNHS 2023).

		Diabetes (N = 880)	Without Diabetes (N = 880)	
Variable	Categories	N (%)	N (%)	*p*-Value
Age groups	35–59 years	174 (19.8)	174 (19.8)	NA
	60–69 years	284 (32.3)	284 (32.3)	
	70–79 years	270 (30.7)	270 (30.7)	
	80 years or older	152 (17.3)	152 (17.3)	
Educational level	Ended before 13 years of age	253 (32.8)	254 (31.6)	0.273
	Ended from 13 to 16 years	202 (26.2)	188 (23.4)	
	Ended from 16 to 18 or vocational training	195 (25.3)	203 (25.2)	
	Completed university studies	122 (15.8)	160 (19.9)	
Employment status	Working	196 (22.3)	225 (25.6)	0.082
	Unemployed	52 (5.9)	41 (4.7)	
	Retired or disabled	630 (71.7)	609 (69.3)	
	Housework or studying	1 (0.1)	4 (0.5)	
Monthly household income	Less than 1600 euros	207 (24.4)	229 (26.9)	0.727
	From 1650 to 2300 euros	142(16.7)	127 (14.9)	
	From 2300 to 3800 euros	242 (28.5)	262 (30.8)	
	From 3800 euros onwards	257 (30.3)	233 (27.4)	
Living with a partner	Yes	516 (59.0)	523 (59.8)	0.806
Oslo Social Support Scale	Poor Social Support	44 (5.2)	52 (6.1)	0.763
	Moderate Social Support	312 (36.8)	305 (36.0)	
	Strong Social Support	492 (58.0)	491 (57.9)	
Self-rated health	Very good, good	402 (45.7)	585 (66.5)	<0.001
Cardiovascular diseases	Yes	271 (30.8)	158 (18.0)	<0.001
Musculoskeletal disorders	Yes	410 (46.6)	293 (33.3)	<0.001
Respiratory diseases	Yes	98 (11.1)	76 (8.6)	0.085
Gastrointestinal diseases	Yes	196 (22.3)	150 (17.0)	0.005
Neurological diseases	Yes	58 (6.6)	49 (5.6)	0.371
Malignant tumors	Yes	76 (8.6)	70 (8.0)	0.604
Hypertension	Yes	557(62.5)	381(42.8)	<0.001
Number of chronic conditions	Mean (SD)	1.89 (1.31)	1.34 (1.24)	<0.001
Tobacco use	Currently	151 (17.2)	164 (18.7)	0.003
	Ex smoker	430 (49.0)	362 (41.3)	
	Never smoked	297 (33.8)	351 (40.0)	
Alcohol use	Daily or weekly	368 (42.1)	421 (48.4)	0.001
	Few times a year	206 (23.6)	217 (24.9)	
	Not last year or ever	300 (34.3)	232 (26.7)	
Physically active	Yes	118 (13.6)	176 (20.2)	<0.001
Weight	Normal	188 (22.0)	289 (34.0)	<0.001
	Overweight	418 (48.9)	401 (47.1)	
	Obesity	248 (29.0)	161 (18.9)	
Low emotional well-being (WHO-5 < 50)	Yes	144 (16.4)	102 (11.6)	0.004
Clinically relevant depressive symptoms (PHQ-8 ≥ 10)	Yes	277 (32.7)	212 (24.9)	<0.001
Psychiatric medication use	Yes	180 (20.5)	134 (15.2)	0.005

WHO-5: World Health Organization-Five Well-Being Index. PHQ-8: Patient Health Questionnaire Eight Items. *p* values for difference between participants with diabetes vs. matched participants without diabetes (bivariate conditional logistic regression).

**Table 2:** Sociodemographic, health-related, lifestyle and mental health characteristics among women reporting diabetes and matched controls without diabetes in Spain (SNHS 2023).

		Diabetes (N = 975)	Without Diabetes (N = 975)	
Variable	Categories	N (%)	N (%)	*p*-Value
Age groups	35–59 years	163 (16.7)	163 (16.7)	NA
	60–69 years	186 (19.1)	186 (19.1)	
	70–79 years	317 (32.5)	317 (32.5)	
	80 years or older	309 (31.7)	309 (31.7)	
Educational level	Ended before 13 years of age	361 (47.9)	320 (40.8)	<0.001
	Ended from 13 to 16 years	161 (21.4)	134 (17.1)	
	Ended from 16 to 18 or vocational training	140 (18.6)	179 (22.8)	
	Completed university studies	91 (12.1)	152 (19.4)	
Employment status	Working	118 (12.1)	164 (16.9)	<0.001
	Unemployed	44 (4.5)	26 (2.7)	
	Retired or disabled	675 (69.4)	675 (69.4)	
	Housework or studying	136 (14.0)	107 (11.0)	
Monthly household income	Less than 1600 euros	252 (26.6)	233 (24.8)	0.717
	From 1650 to 2300 euros	167 (17.6)	165 (17.6)	
	From 2300 to 3800 euros	302 (31.9)	303 (32.2)	
	From 3800 euros onwards	227 (23.9)	239 (25.4)	
Living with a partner	Yes	352 (36.5)	354 (36.6)	0.998
Oslo Social Support Scale	Poor Social Support	67 (7.0)	49 (5.2)	0.082
	Moderate Social Support	334 (34.9)	307 (32.7)	
	Strong Social Support	557 (58.1)	582 (62.0)	
Self-rated health	Very good, good	318 (32.6)	506 (51.9)	<0.001
Cardiovascular diseases	Yes	234 (24.0)	143 (14.7)	<0.001
Musculoskeletal disorders	Yes	680 (69.7)	557 (57.1)	<0.001
Respiratory diseases	Yes	154 (15.8)	94 (9.6)	<0.001
Gastrointestinal diseases	Yes	254 (26.1)	216 (22.2)	0.039
Neurological diseases	Yes	188 (19.3)	133 (13.6)	<0.001
Malignant tumors	Yes	113 (11.6)	70 (7.2)	<0.001
Hypertension	Yes	647 (65.2)	423 (42.6)	<0.001
Number of chronic conditions	Mean, SD	2.32 (1.42)	1.67 (1.35)	<0.001
Tobacco use	Currently	86 (8.8)	76 (7.8)	0.301
	Ex smoker	157 (16.2)	178 (18.3)	
	Never smoked	729 (75.0)	717 (73.8)	
Alcohol use	Daily or weekly	96 (9.9)	189 (19.6)	<0.001
	Few times a year	201 (20.8)	262 (27.1)	
	Not last year or ever	669 (69.3)	515 (53.3)	
Physically active	Yes	85 (8.8)	149 (15.5)	<0.001
Weight	Normal	274 (30.8)	396 (44.0)	<0.001
	Overweight	345 (38.7)	361 (40.1)	
	Obesity	272 (30.5)	143 (15.9)	
Low emotional well-being (WHO-5 < 50)	Yes	281 (28.8)	211 (21.6)	<0.001
Clinically relevant depressive symptoms (PHQ-8 ≥ 10)	Yes	507 (54.4)	376 (40.2)	<0.001
Psychiatric medication use	Yes	385 (39.5)	294 (30.2)	<0.001

WHO-5: World Health Organization-Five Well-Being Index. PHQ-8: Patient Health Questionnaire Eight Items. *p* values for difference between participants with diabetes vs. matched participants without diabetes (bivariate conditional logistic regression). Mental health outcomes among men according to study variables.

**Table 3:** Prevalence of low emotional well-being (WHO-5 < 50) and depressive symptoms (PHQ-8 ≥ 10) by sociodemographic characteristics among men reporting diabetes and matched controls without diabetes in Spain (Spanish National Health Survey 2023).

		Low Emotional Well-Being (WHO-5 < 50)			Depressive Symptoms (PHQ-8 ≥ 10)			Psychiatric Medication Use		
		Diabetes	Without Diabetes		Diabetes	Without Diabetes		Diabetes	Without Diabetes	
Variable	Categories	N (%)	N (%)	*p*-Value	N (%)	N (%)	*p*-Value	N (%)	N (%)	*p*-Value
Age group ^a,c,d,e^	35–59 years	36 (20.7)	20 (11.5)	0.024	57 (33.9)	46 (27.2)	0.142	32 (18.4)	20 (11.5)	0.080
	60–69 years	39 (13.7)	30 (10.6)	0.027	74 (26.9)	57 (20.7)	0.001	47 (16.5)	40 (14.1)	0.018
	70–79 years	33 (12.2)	28 (10.4)	0.083	79 (30.3)	62 (23.8)	<0.001	53 (19.6)	44 (16.3)	<0.001
	80 years or older	36 (23.7)	24 (15.8)	0.015	67 (46.9)	47 (32.6)	0.004	48 (31.6)	30 (19.7)	0.139
Educational level	Ended before 13 years of age	45 (17.8)	30 (11.8)	0.055	88 (36.8)	71 (28.9)	0.231	47 (18.6)	38 (15.0)	0.513
	Ended from 13 to 16 years	32 (15.8)	21 (11.2)	0.226	57 (28.8)	52 (28.1)	0.758	33 (16.3)	30 (16.0)	0.506
	Ended from 16 to 18 or vocational training	28 (14.4)	20 (9.9)	0.533	58 (30.5)	44 (22.6)	0.332	45 (23.1)	25 (12.3)	0.019
	Completed university studies	16 (13.1)	17 (10.6)	0.763	34 (28.8)	29 (18.8)	0.796	26 (21.3)	25 (15.6)	0.763
Employment status ^c,e,f^	Working	25 (12.8)	24 (10.7)	0.998	44 (23.0)	47 (21.5)	0.039	22 (11.2)	21 (9.3)	0.250
	Unemployed	14 (26.9)	4 (9.8)	0.341	22 (43.1)	14 (35.0)	0.423	13 (25.0)	5 (12.2)	0.121
	Retired or disabled	104 (16.5)	74 (12.2)	0.007	209 (34.7)	151 (25.7)	<0.001	145 (23.0)	108 (17.7)	0.001
	Housework or studying	1 (100.0)	0 (0.0)	0.178	1 (100.0)	0 (0.0)	0.067	0 (0.0)	0 (0.0)	0.099
Monthly household income	Less than 1600 euros	31 (15.1)	24 (10.6)	0.998	67 (33.7)	57 (25.8)	0.134	52 (25.4)	45 (19.8)	0.442
	From 1650 to 2300 euros	23 (16.8)	12 (9.7)	0.998	39 (29.1)	31 (25.4)	0.706	25 (18.2)	20 (16.1)	0.657
	From 2300 to 3800 euros	35 (14.5)	36 (13.9)	0.763	71 (31.0)	59 (23.9)	0.998	45 (18.6)	38 (14.7)	0.618
	From 3800 euros onwards	49 (19.4)	24 (10.4)	0.068	89 (36.3)	58 (26.4)	0.396	53 (20.9)	25 (10.9)	0.619
Living with a partner	Yes	82 (15.9)	54 (10.3)	0.011	151 (30.4)	121 (24.0)	<0.001	99 (19.2)	70 (13.4)	0.006
	No	59 (16.5)	47 (13.4)	0.033	123 (35.5)	89 (26.2)	<0.001	79 (22.1)	63 (17.9)	0.026
Oslo Social Support Scale ^a,b,c,d^	Poor Social Support	15 (34.1)	11 (21.2)	0.155	26 (60.5)	19 (38.0)	0.571	13 (29.5)	12 (23.1)	0.472
	Moderate Social Support	51 (16.3)	38 (12.5)	0.072	114 (38.3)	88 (29.8)	<0.001	60 (19.2)	43 (14.1)	0.001
	Strong Social Support	67 (13.6)	48 (9.8)	0.133	130 (27.1)	99 (20.8)	<0.001	97 (19.7)	77 (15.7)	0.023

WHO-5: World Health Organization-Five Well-Being Index. PHQ-8: Patient Health Questionnaire Eight Items. *p* values for difference between participants with diabetes vs. matched participants without diabetes (bivariate conditional logistic regression). ^a^ Significant association (*p* < 0.05) among diabetic subjects with low emotional well-being (WHO-5 < 50). ^b^ Significant association (*p* < 0.05) among subjects without diabetes with low emotional well-being (WHO-5 < 50). ^c^ Significant association (*p* < 0.05) among diabetic subjects with depressive symptoms. ^d^ Significant association (*p* < 0.05) among subjects without diabetes with depressive symptoms. ^e^ Significant association (*p* < 0.05) among diabetic subjects with psychiatric medication use. ^f^ Significant association (*p* < 0.05) among subjects without diabetes with psychiatric medication use. Association within each study subgroup (diabetes/no diabetes) was assessed with bivariate unconditional logistic regression.

**Table 4:** Prevalence of low emotional well-being (WHO-5 < 50) and depressive symptoms (PHQ-8 ≥ 10) by health-related and lifestyle characteristics among men reporting diabetes and matched controls without diabetes in Spain (Spanish National Health Survey 2023).

		Low Emotional Well-Being (WHO-5 < 50)			Depressive Symptoms (PHQ-8 ≥ 10)			Psychiatric Medication Use		
		Diabetes	Without Diabetes		Diabetes	Without Diabetes		Diabetes	Without Diabetes	
Variable	Categories	N (%)	N (%)	*p*-Value	N (%)	N (%)	*p*-Value	N (%)	N (%)	*p*-Value
Self-rated health ^a,b,c,d,e,f^	Very good, good	20 (5.0)	25 (4.3)	0.447	68 (17.1)	86 (15.1)	0.206	44 (10.9)	53 (9.1)	0.032
	Fair, bad, very bad	124 (25.9)	77 (26.1)	0.641	209 (46.4)	126 (45.2)	0.195	136 (28.5)	81 (27.5)	0.112
Cardiovascular diseases ^a,c,e,f^	Yes	64 (23.6)	20 (12.7)	0.030	110 (42.1)	46 (31.1)	0.288	81 (29.9)	43 (27.2)	0.152
	No	80 (13.1)	82 (11.4)	0.007	167 (28.5)	166 (23.6)	<0.001	99 (16.3)	91 (12.6)	<0.001
Musculoskeletal disorders ^a,b,c,d,e,f^	Yes	83 (20.2)	52 (17.7)	0.060	154 (39.3)	95 (34.1)	0.007	106 (25.9)	69 (23.5)	0.029
	No	61 (13.0)	50 (8.5)	0.156	123 (27.0)	117 (20.5)	0.001	74 (15.7)	65 (11.1)	0.080
Respiratory diseases ^a,b,c,d,e,f^	Yes	30 (30.6)	18 (23.7)	0.423	49 (53.3)	36 (48.6)	0.530	33 (33.7)	19 (25.0)	0.998
	No	114 (14.6)	84 (10.4)	0.008	228 (30.2)	176 (22.7)	<0.001	147 (18.8)	115 (14.3)	<0.001
Gastrointestinal diseases ^a,b,c,d,e,f^	Yes	55 (28.1)	32 (21.3)	0.889	83 (44.6)	52 (36.6)	0.163	68 (34.7)	32 (21.3)	0.032
	No	89 (13.0)	70 (9.6)	0.003	194 (29.3)	160 (22.6)	<0.001	112 (16.4)	102 (14.0)	0.006
Neurological diseases ^a,b,c,d,e,f^	Yes	23 (39.7)	17 (34.7)	0.618	36 (69.2)	21 (50.0)	0.410	28 (48.3)	21 (42.9)	0.763
	No	121 (14.7)	85 (10.2)	<0.001	241 (30.3)	191 (23.6)	<0.001	152 (18.5)	113 (13.6)	<0.001
Malignant tumors ^b,f^	Yes	14 (18.4)	14 (20.0)	0.327	24 (33.8)	23 (34.3)	0.423	19 (25.0)	21 (30.0)	0.484
	No	130 (16.2)	88 (10.9)	<0.001	253 (32.6)	189 (24.1)	<0.001	161 (20.0)	113 (14.0)	<0.001
Hypertension ^c,d,f^	Yes	101 (18.1)	42 (11.0)	0.006	196 (36.6)	107 (28.9)	0.101	117 (21.0)	72 (18.9)	0.102
	No	43(13.39	60 (12.0)	0.884	81 (26.0)	105 (21.9)	0.529	63 (19.5)	62 (12.4)	0.606
Number of chronic conditions, ^a,b,c,d,e,f^	Mean, SD	2.56 (1.41)	1.91 (1.39)	<0.001	2.35 (1.45)	1.79 (1.34)	<0.001	2.51 (1.41)	2.07 (1.44)	0.006
Tobacco use	Currently	31 (20.5)	17 (10.4)	0.178	55 (37.7)	40 (25.2)	0.069	30 (19.9)	29 (17.7)	0.207
	Ex smoker	71 (16.5)	40 (11.0)	0.018	139 (33.3)	85 (24.1)	0.042	96 (22.3)	62 (17.1)	0.006
	Never smoked	42 (14.1)	45 (12.8)	0.024	83 (29.4)	87 (25.9)	0.001	53 (17.8)	43 (12.3)	0.003
Alcohol use ^c,d,e^	Daily or weekly	55 (14.9)	44 (10.5)	0.457	88 (24.7)	87 (21.2)	0.125	65 (17.7)	65 (15.4)	0.161
	Few times a year	29 (14.1)	21 (9.7)	0.400	68 (34.3)	47 (22.7)	0.024	37 (18.0)	28 (12.9)	0.015
	Not last year or ever	60 (20.0)	36 (15.5)	0.161	121 (42.0)	77 (34.4)	0.005	77 (25.7)	41 (17.7)	0.039
Physically active ^c,d^	Yes	12 (10.2)	21 (11.9)	0.998	18 (15.5)	29 (16.8)	0.099	19 (16.1)	24 (13.6)	0.763
	No	126 (16.8)	79 (11.4)	<0.001	251 (34.8)	183 (27.3)	<0.001	157 (20.9)	109 (15.7)	<0.001
Weight	Normal	38 (20.2)	37 (12.8)	0.194	53 (30.3)	69 (25.0)	0.055	47 (25.0)	44 (15.2)	0.300
	Overweight	61 (14.6)	40 (10.0)	0.061	124 (30.1)	90 (23.0)	0.003	75 (17.9)	58 (14.5)	0.041
	Obesity	40 (16.1)	18 (11.2)	0.024	87 (36.6)	44 (28.0)	0.046	52 (21.0)	28 (17.4)	0.091

WHO-5: World Health Organization-Five Well-Being Index. PHQ-8: Patient Health Questionnaire Eight Items. *p* values for difference between participants with diabetes vs. matched participants without diabetes (bivariate conditional logistic regression). ^a^ Significant association (*p* < 0.05) among diabetic subjects with low emotional well-being (WHO-5 < 50). ^b^ Significant association (*p* < 0.05) among subjects without diabetes with low emotional well-being (WHO-5 < 50). ^c^ Significant association (*p* < 0.05) among diabetic subjects with depressive symptoms. ^d^ Significant association (*p* < 0.05) among subjects without diabetes with depressive symptoms. ^e^ Significant association (*p* < 0.05) among diabetic subjects with psychiatric medication use. ^f^ Significant association (*p* < 0.05) among subjects without diabetes with psychiatric medication use. Association within each study subgroup (diabetes/no diabetes) was assessed with bivariate unconditional logistic regression.

**Table 5:** Prevalence of low emotional well-being (WHO-5 < 50) and depressive symptoms (PHQ-8 ≥ 10) by sociodemographic characteristics among women reporting diabetes and matched controls without diabetes in Spain (Spanish National Health Survey 2023).

		Low Emotional Well-Being (WHO-5 < 50)			Depressive Symptoms (PHQ-8 ≥ 10)			Psychiatric Medication Use		
		Diabetes	Without Diabetes		Diabetes	Without Diabetes		Diabetes	Without Diabetes	
Variable	Categories	N (%)	N (%)	*p*-Value	N (%)	N (%)	*p*-Value	N (%)	N (%)	*p*-Value
Age group ^a,b,c,d,e,f^	35–59 years	46 (28.2)	29 (17.8)	0.032	77 (49.0)	44 (28.0)	<0.001	44 (27.0)	26 (16.0)	0.016
	60–69 years	35 (18.8)	21 (11.3)	0.027	84 (46.2)	50 (28.1)	0.001	63 (33.9)	41 (22.0)	0.018
	70–79 years	75 (23.7)	58 (18.3)	0.083	155 (50.8)	107 (34.9)	<0.001	131 (41.3)	83 (26.2)	<0.001
	80 years or older	125 (40.5)	103 (33.3)	0.015	191 (66.3)	175 (59.5)	0.004	147 (47.6)	144 (46.6)	0.139
Educational level ^a,b,d,e,f^	Ended before 13 years of age	101 (28.0)	79 (24.7)	0.055	181 (52.6)	139 (45.6)	0.231	145 (40.2)	114 (35.6)	0.513
	Ended from 13 to 16 years	38 (23.6)	22 (16.4)	0.226	79 (50.6)	35 (27.3)	0.758	63 (39.1)	37 (27.6)	0.506
	Ended from 16 to 18 or vocational training	44 (31.4)	32 (17.9)	0.533	77 (56.6)	65 (37.8)	0.332	57 (40.7)	38 (21.2)	0.019
	Completed university studies	12 (13.2)	19 (12.5)	0.763	39 (43.8)	35 (23.6)	0.796	20 (22.0)	23 (15.1)	0.763
Employment status ^a,d,e,f^	Working	20 (16.9)	24 (14.6)	0.998	51 (44.7)	43 (27.2)	0.039	21 (17.8)	25 (15.2)	0.250
	Unemployed	16 (36.4)	4 (15.4)	0.341	21 (50.0)	6 (23.1)	0.423	11 (25.0)	4 (15.4)	0.343
	Retired or disable	206 (30.5)	157 (23.3)	0.007	366 (56.7)	282 (43.7)	<0.001	304 (45.0)	230 (34.1)	0.001
	Housework or studying	39 (28.7)	25 (23.4)	0.178	67 (51.9)	44 (42.3)	0.067	49 (36.0)	35 (32.7)	0.099
Monthly household income	Less than 1600 euros	73 (29.6)	44 (19.3)	0.350	120 (50.4)	85 (38.6)	0.435	100 (40.5)	79 (34.6)	0.842
	From 1650 to 2300 euros	46 (28.0)	34 (20.7)	0.998	83 (54.2)	59 (36.9)	0.039	64 (39.0)	41 (25.0)	0.410
	From 2300 to 3800 euros	83 (28.1)	66 (22.0)	0.425	161 (56.7)	113 (39.1)	0.001	123 (41.7)	82 (27.3)	0.056
	From 3800 euros onwards	65 (28.9)	55 (23.5)	0.827	121 (56.8)	102 (46.6)	0.183	81 (36.0)	77 (32.9)	0.842
Living with a partner ^b,c,d,f^	Yes	90 (25.6)	59 (16.7)	0.011	162 (47.6)	120 (34.9)	<0.001	126 (35.8)	93 (26.3)	0.006
	No	190 (31.0)	151 (24.6)	0.033	339 (58.2)	253 (43.2)	<0.001	256 (41.8)	199 (32.4)	0.026
Oslo Social Support Scale ^a,b,c,d,e^	Poor Social Support	42 (62.7)	16 (32.7)	0.001	47 (79.7)	27 (56.3)	0.571	31 (46.3)	11 (22.4)	0.008
	Moderate Social Support	108 (32.3)	71 (23.1)	0.072	201 (63.0)	139 (47.3)	<0.001	145 (43.4)	82 (26.7)	0.001
	Strong Social Support	124 (22.3)	111 (19.1)	0.133	251 (46.4)	198 (35.0)	<0.001	200 (35.9)	192 (33.0)	0.023

WHO-5: World Health Organization-Five Well-Being Index. PHQ-8: Patient Health Questionnaire Eight Items. *p* values for difference between participants with diabetes vs. matched participants without diabetes (bivariate conditional logistic regression). ^a^ Significant association (*p* < 0.05) among diabetic subjects with low emotional well-being (WHO-5 < 50). ^b^ Significant association (*p* < 0.05) among subjects without diabetes with low emotional well-being (WHO-5 < 50). ^c^ Significant association (*p* < 0.05) among diabetic subjects with depressive symptoms. ^d^ Significant association (*p* < 0.05) among subjects without diabetes with depressive symptoms. ^e^ Significant association (*p* < 0.05) among diabetic subjects with psychiatric medication use. ^f^ Significant association (*p* < 0.05) among subjects without diabetes with psychiatric medication use. Association within each study subgroup (diabetes/no diabetes) was assessed with bivariate unconditional logistic regression.

**Table 6:** Prevalence of low emotional well-being (WHO-5 < 50) and depressive symptoms (PHQ-8 ≥ 10) by health-related and lifestyle characteristics among women reporting diabetes and matched controls without diabetes in Spain (Spanish National Health Survey 2023).

		Low Emotional Well-Being (WHO-5 < 50)			Depressive Symptoms (PHQ-8 ≥ 10)			Psychiatric Medication Use		
		Diabetes	Without Diabetes		Diabetes	Without Diabetes		Diabetes	Without Diabetes	
Variable	Categories	N (%)	N (%)	*p*-Value	N (%)	N (%)	*p*-Value	N (%)	N (%)	*p*-Value
Self-rated health ^a,b,c,d,e,f^	Very good, good	26 (8.2)	37 (7.3)	0.447	91 (29.4)	107 (21.8)	0.206	69 (21.7)	71 (14.0)	0.032
	Fair, bad, very bad	255 (38.8)	174 (37.1)	0.641	416 (66.9)	269 (60.4)	0.195	316 (48.1)	223 (47.5)	0.112
Cardiovascular diseases ^a,b,c,d,e,f^	Yes	99 (42.3)	51 (35.7)	0.030	143 (65.3)	78 (59.1)	0.288	122 (52.1)	73 (51.0)	0.152
	No	182 (24.6)	160 (19.2)	0.007	364 (51.1)	298 (37.1)	<0.001	263 (35.5)	221 (26.6)	<0.001
Musculoskeletal disorders ^a,b,c,d,e,f^	Yes	234 (34.4)	155 (27.8)	0.060	393 (60.7)	275 (51.5)	0.007	317 (46.6)	236 (42.4)	0.029
	No	47 (15.9)	56 (13.4)	0.156	114 (40.0)	101 (25.1)	0.001	68 (23.1)	58 (13.9)	0.080
Respiratory diseases ^a,c,d,f^	Yes	62 (40.3)	24 (25.5)	0.423	94 (63.1)	48 (52.7)	0.530	71 (46.1)	41 (43.6)	0.998
	No	219 (26.7)	187 (21.2)	0.008	413 (52.7)	328 (38.8)	<0.001	314 (38.2)	253 (28.7)	<0.001
Gastrointestinal diseases ^a,b,c,d,e,f^	Yes	104 (40.9)	81 (37.5)	0.889	166 (69.5)	110 (54.5)	0.163	139 (54.7)	101 (46.8)	0.032
	No	177 (24.5)	130 (17.1)	0.003	341 (49.2)	266 (36.2)	<0.001	246 (34.1)	193 (25.4)	0.006
Neurological diseases ^a,b,c,d,e,f^	Yes	82 (43.6)	71 (53.4)	0.618	115 (67.6)	81 (66.9)	0.410	114 (60.6)	75 (56.4)	0.763
	No	199 (25.3)	140 (16.6)	<0.001	392 (51.4)	295 (36.2)	<0.001	271 (34.4)	219 (26.0)	<0.001
Malignant tumors ^a,b,c,e,f^	Yes	42 (37.2)	29 (41.4)	0.327	76 (69.7)	34 (51.5)	0.423	63 (55.8)	31 (44.3)	0.484
	No	239 (27.7)	182 (20.1)	<0.001	431 (52.4)	342 (39.3)	<0.001	322 (37.4)	263 (29.1)	<0.001
Hypertension ^a,b,d,e,f^	Yes	206 (32.1)	118 (28.0)	0.082	345 (56.7)	197 (49.1)	0.076	278 (43.4)	173 (41.0)	0.556
	No	75 (22.5)	93 (16.8)	0.051	162 (50.2)	179 (33.5)	0.002	107 (32.0)	121 (21.9)	0.009
Number of chronic conditions ^a,b,c,d,e,f^	Mean, SD	2.95 (1.42)	2.51 (1.48)	0.001	2.63 (1.43)	2.19 (1.41)	<0.001	2.86 (1.39)	2.48 (1.32)	<0.001
Tobacco use ^d^	Currently	22 (25.6)	14 (18.4)	0.178	50 (58.1)	17 (23.6)	0.069	36 (41.9)	19 (25.0)	0.207
	Ex smoker	47 (29.9)	29 (16.3)	0.018	69 (46.9)	56 (32.7)	0.042	64 (40.8)	47 (26.4)	0.006
	Never smoked	210 (28.8)	167 (23.3)	0.024	387 (55.4)	303 (43.9)	0.001	284 (39.0)	228 (31.8)	0.003
Alcohol use ^a,b,c,d,f^	Daily or weekly	22 (22.9)	29 (15.3)	0.457	47 (51.1)	49 (26.8)	0.125	30 (31.3)	38 (20.1)	0.161
	Few times a year	41 (20.4)	41 (15.6)	0.400	91 (46.4)	89 (35.2)	0.024	74 (36.8)	65 (24.8)	0.015
	Not last year or ever	214 (32.0)	139 (27.0)	0.161	366 (57.3)	238 (48.3)	0.005	277 (41.4)	189 (36.7)	0.039
Physically active ^a,b,c,d,f^	Yes	8 (9.4)	17 (11.4)	0.998	27 (32.1)	36 (24.8)	0.099	31 (36.5)	34 (22.8)	0.763
	No	269 (30.5)	189 (23.2)	<0.001	478 (56.7)	337 (43.0)	<0.001	351 (39.8)	258 (31.7)	<0.001
Weight ^a^	Normal	67 (24.5)	83 (21.0)	0.194	131 (50.2)	152 (39.9)	0.055	96 (35.0)	121 (30.6)	0.300
	Overweight	84 (24.3)	70 (19.4)	0.061	173 (51.5)	144 (40.4)	0.003	136 (39.4)	104 (28.8)	0.041
	Obesity	95 (34.9)	37 (25.9)	0.024	157 (59.5)	63 (46.0)	0.046	115 (42.3)	45 (31.5)	0.091

WHO-5: World Health Organization-Five Well-Being Index. PHQ-8: Patient Health Questionnaire Eight Items. *p* values for difference between participants with diabetes vs. matched participants without diabetes (bivariate conditional logistic regression). ^a^ Significant association (*p* < 0.05) among diabetic subjects with low emotional well-being (WHO-5 < 50). ^b^ Significant association (*p* < 0.05) among subjects without diabetes with low emotional well-being (WHO-5 < 50). ^c^ Significant association (*p* < 0.05) among diabetic subjects with depressive symptoms. ^d^ Significant association (*p* < 0.05) among subjects without diabetes with depressive symptoms. ^e^ Significant association (*p* < 0.05) among diabetic subjects with psychiatric medication use. ^f^ Significant association (*p* < 0.05) among subjects without diabetes with psychiatric medication use. Association within each study subgroup (diabetes/no diabetes) was assessed with bivariate unconditional logistic regression.

**Table 7:** Conditional logistic regression models for low emotional well-being (WHO-5 < 50), clinically relevant depressive symptoms (PHQ-8 ≥ 10) and psychiatric medication use among men and women reporting diabetes and matched controls without diabetes in Spain (Spanish National Health Survey 2023).

	Low Emotional Well-Being (WHO-5 < 50)		Clinically Relevant Depressive Symptoms (PHQ-8 ≥ 10)		Psychiatric Medication Use	
	Men	Women	Men	Women	Men	Women
Model 1. OR (95% CI)	1.51	1.48	1.52	1.93	1.42	1.56
	(1.14–2.00)	(1.20–1.82)	(1.22–1.90)	(1.58–2.36)	(1.11–1.82)	(1.28–1.91)
Model 2. aOR (95% CI)	1.48	1.43	1.31	1.38	1.34	1.35
	(1.03–2.13)	(1.14–1.78)	(1.02–1.69)	(1.08–1.77)	(1.02–1.75)	(1.06–1.73)

OR: odds ratio. aOR: adjusted odds ratio. CI: confidence interval. Dependent variables: low emotional well-being (WHO-5 < 50), clinically relevant depressive symptoms (PHQ-8 ≥ 10), and prescribed psychiatric medication use. The principal exposure was diabetes status, with participants without diabetes as the reference category. Model 1: Unadjusted conditional logistic regression model. Model 2: Adjusted for sociodemographic characteristics, perceived social support, chronic health conditions, and lifestyle-related factors. Analyses were stratified by sex.

## Data Availability

Data of the Spanish National Health Survey 2023 can be freely downloaded from https://www.sanidad.gob.es/estadisticas/microdatos.do (accessed on 12 June 2026).

## Supplementary Materials

The following supporting information can be downloaded at https://www.mdpi.com/article/10.3390/healthcare14172691/s1, Table S1: Definition of study variables according to the questions included in the Spanish National Health Interview Survey 2023; Table S2: Sociodemographic, health-related, lifestyle and mental health characteristics among participants with diabetes excluded for missing data on mental health outcomes and participant with diabetes and complete data on mental outcomes participating in the SNHS 2023; Figure S1: Factors associated with low emotional well-being in men with diabetes; Figure S2: Factors associated with depressive symptoms in men with diabetes; Figure S3: Factors associated with psychiatric medication use in men with diabetes; Figure S4: Factors associated with low emotional well-being in women with diabetes; Figure S5: Factors associated with depressive symptoms in women with diabetes; Figure S6: Factors associated with psychiatric medication use in women with diabetes.

## Abbreviations

The following abbreviations are used in this manuscript:

aOR	Adjusted odds ratio
SNHS 2023	Spanish National Health Survey
SMH	Spanish Ministry of Health
WHO-5	World Health Organization-Five Well-Being Index
PHQ-8	Patient Health Questionnaire
OR	Odds ratio
OSSS-3	Oslo Social Support Scale
BMI	Body mass index
