# Supplementary material for: Mental Health Among Adults with Diabetes in Spain: A Population-Based Matched Study of Emotional Well-Being, Depressive Symptoms, and Psychiatric Medication Use
Source: Healthcare (Basel). 2026 Aug 24;14(17):2691. doi: 10.3390/healthcare14172691 (PMC13565456; doi:10.3390/healthcare14172691)

Table S1. Definition of study variables according to the questions included in the Spanish National Health Interview Survey 2023.

Questions	Description and answer	Variables name	Categories
Has your doctor told you that you are suffering from diabetes?	Yes or no	Diabetes	1. Yes 2. No
WHO-5 Well-Being Index	Emotional well-being was assessed using the WHO-5 Well-Being Index. The Ministry of Health provided a transformed score ranging from 0 to 100, with higher values indicating better emotional well-being.	Low emotional well-being	1. Yes: WHO-5 score <50<br>2. No: WHO-5 score ≥50
PHQ-8 depressive symptom severity	Depressive symptom severity was assessed using the Patient Health Questionnaire-8 (PHQ-8). Moderate-to-severe depressive symptoms were considered present when PHQ-8 score was ≥10.	Depressive symptom severity	1. Yes: PHQ-8 ≥10. Clinically relevant depressive symptoms<br>2. No PHQ-8 <10. No depressive symptoms
- Next I am going to read you a list of types of medications, Please tell me which one of them you have taken in the last 2 weeks?<br>- Was it prescribed by the doctor?	A list of 23 medications is read to the person interviewed. For those medications with an affirmative answer in the first question, the second are completed consecutively for each specific medication.<br>1. Tranquilizers sedatives or “sleeping pills”,<br>2. Antidepressants	Psychiatric medications use	1. Yes: Those who answered “yes” to both question for options 1 and/or 2<br>2. No: Answered “no” to one or both questions for options 1 and 2
Which is your sex?	1. Man<br>2. Woman	Sex	1. Man<br>2. Woman
How old are you?	Age in years	Age groups	35-59 years<br>60-69 years<br>70-79 years<br>80 years or older

Questions	Description and answer	Variables name	Categories
What level of education have you completed?	1. Does not know how to read or write<br>2. Incomplete primary education<br>3. Complete primary education<br>4. First stage of Secondary Education, with or without a qualification<br>5. Elementary Spanish Upper Secondary Education<br>6. Upper secondary education<br>7. Intermediate vocational training or equivalent<br>8. Advanced vocational training or equivalent<br>9. University studies or equivalent<br>10. University (master, PhD….)	Educational level	1. Ended before 13 years of age: Options 1 to 3<br>2. Ended from 13 to 16 years: Options 4 and 5<br>3. Ended from 16 to 18 or vocational training. Options 6 to 8.<br>4 Completed university studies: Options 9 and 10
Which is your employment status?	1. Working<br>2. Unemployed<br>3. Retired<br>4. Studying<br>5. Unable to work<br>6. Housework<br>7. Other	Employment status	1. Working. Option 1<br>2. Unemployed Option 2<br>3. Retired, or disable Options 3 and 5<br>4. Housework or studying. Options 4 and 6
Could you tell us which of the following ranges best represents your entire household's net monthly income, after deductions for taxes, Social Security, etc.?	1. Less than 1100 euros<br>2. From 1100 to less than 1650 euros<br>3. From 1650 to less than 2300 euros<br>4. From 2300 to less than 3800euros<br>From 3800 euros onwards	Monthly household income	1. Less than 1650 euros. Options 1 and 2<br>2.From 1650 to less than 2300 euros. Option 3<br>3. .From 2300 to less than 3800 euros. Option 4<br>4. From 3800 euros onwards. Option 5
Of the following options, which describes your current situation?	1. Living with your spouse<br>2. Living with a common-law partner<br>3. Not living together as a couple	Living with a partner	1. Yes: Option 1 and 2<br>2. No: option 3
**The Oslo Social Support Scale. Question 1**	1. None	Social support	

Questions	Description and answer	Variables name	Categories
In case you have a serious personal problem of any kind, how many people close to you could you count on?	2. 1 or 2 people<br>3. From 3 to 5 people<br>4. More than 5 people		The sum score ranges from 3 to 14, with high values representing strong levels and low values representing poor levels of social support.<br>We defined three categories*<br>1. 3–8 poor social support.<br>2. 9–11 moderate social support.<br>3. 12–14 strong social support.
**The Oslo Social Support Scale. Question 2**<br>To what extent are other people interested in what happens to you? We are referring to both family members and people who are not members of your family.	1. None<br>2. Little<br>3. Not much or little<br>4. Some<br>5. A lot		
**The Oslo Social Support Scale. Question 3**<br>How easy it is to get help from neighbors in case of need	1. Very difficult<br>2. Difficult<br>3. It is possible<br>4. Easy<br>5. Very easy		
In the past twelve months, how is your perception of your general health status?	1. Very good<br>2. Good<br>3. Fair<br>4. Bad<br>5. Very bad	Self-rated health	1. Very good/good: Options 1 and 2<br>2. Fair/poor/very poor: Options 3 to 5
Has your doctor told you that you are suffering from high blood pressure?	Yes or no	Hypertension	1. Yes 2. No
"Has your doctor told you that you are suffering from…?"<br>• Myocardial infarction<br>• Angina/coronary.<br>• Heart failure<br>• Other heart diseases<br>• Stroke	Respondents reporting any of the listed cardiovascular conditions were classified as having a physician diagnosed heart diseases and stroke	Cardiovascular diseases	1. Yes (any item = Yes)<br>2. No (all items = No)
"Has your doctor told you that you are suffering from…?"<br>• Osteoarthritis (excluding arthritis);<br>• Chronic cervical back pain<br>• Chronic lumbar back pain<br>• Osteoporosis	Respondents reporting any of the listed musculoskeletal conditions were classified as having a physician diagnosed musculoskeletal disorders	Musculoskeletal disorders	1. Yes (any item = Yes)<br>2. No (all items = No)

Questions	Description and answer	Variables name	Categories
"Has your doctor told you that you are suffering from…?"<br>• Asthma<br>Chronic bronchitis/emphysema/COPD	Respondents reporting any of the listed respiratory conditions were classified as having respiratory disease	Respiratory diseases	1. Yes (any item = Yes)<br>2. No (all items = No)
"Has your doctor told you that you are suffering from…?"<br>• Gastric or duodenal ulcer<br>• Chronic constipation<br>Cirrhosis/liver dysfunction	Respondents reporting any of the listed gastrointestinal conditions were classified as having chronic gastrointestinal disorder	Gastrointestinal diseases	1. Yes (any item = Yes)<br>2. No (all items = No)
"Has your doctor told you that you are suffering from…?"<br>• Alzheimer's disease or other dementias.<br>• Migraine or frequent headache	Respondents reporting any of the listed neurological conditions were classified as having neurological disorder	Neurological diseases	1. Yes (any item = Yes)<br>2. No (all items = No)
Has your doctor told you that you are suffering from malignant tumors?	Yes or no	Malignant tumors	1. Yes 2. No
Number of chronic conditions	It was calculated by adding the presence of the seven chronic diseases described above	Number of chronic conditions	Used as continuous variable (0-7)
Could you tell me if you smoke?	1. Yes, I smoke daily<br>2. Yes, I smoke, but not daily<br>3. I don't currently smoke but have smoked before<br>4. I neither smoke nor have I ever smoked regularly	Tobacco use	1. Currently Options 1 and 2<br>2. Ex-smoker: Options 3<br>3. Never smoked. Option 4
During the past 12 months, how often have you had alcoholic beverages of any kind (i.e. beer, wine, spirits, distilled and mixed drinks, or other alcoholic beverages)?	1. Daily or almost daily<br>2. 5-6 days per week<br>3. 3-4 days per week<br>4. 1-2 days per week<br>5. 2-3 days in a month<br>6. Once a month<br>7. Less than once a month<br>8. Not in the last 12 months have I stopped drinking<br>9. Never or just a few sips to taste it throughout life	Alcohol use	1. Daily or weekly Options 1 to 4<br>2. Few times a year Options 5 to 7<br>3 Not last year or ever. Options 8 and 9

**Questions**	**Description and answer**	**Variables name**	**Categories**
Which of these possibilities best describes how often you do some physical activity in your free time?	1. I don't exercise. I occupy my free time almost completely sedentary.<br>2. I do some occasional physical or sports activities<br>3. I do physical activity several times a month<br>4. I do sports or physical training several times a week	Physically active	1. No: Option 1<br>2. Yes: Option 2 to 4
1. Could you tell me how tall you are, approximately, without shoes?<br>2. Could you tell me your weight, approximately, without shoes and clothes?	Body mass index is calculated with the formulae:<br>Weight in kg/ (Height in meters)^2	Body mass index	1. Normal (<25)<br>2. Overweight (25-29.9)<br>3.Obesity (≥30)

*5.Kocalevent RD, Berg L, Beutel ME, Hinz A, Zenger M, Härter M, Nater U, Brähler E. Social support in the general population: standardization of the Oslo social support scale (OSSS-3). BMC Psychol. 2018 Jul 17;6(1):31. doi: 10.1186/s40359-018-0249-9. PMID: 30016997; PMCID: PMC6050647.

Table S2. Sociodemographic, health-related, lifestyle and mental health characteristics among participants with diabetes excluded for missing data on mental health outcomes and participant with diabetes and complete data on mental outcomes participating in the SNHS 2023

		Excluded	Included	p-value
Age, mean (SD)		74.9(14.26)	70.2(13.4)	0.001
Age groups	35-59 years	13 (15.3%)	332 (17.6%)	0.003
	60-69 years	13 (15.3%)	468 (24.8%)	
	70-79 years	21 (24.7%)	581 (30.8%)	
	>=80 years	38 (44.7%)	505 (26.8%)	
Sex	Women	50 (58.1%)	1,006 (52.5%)	0.310
Educational level	Ended before 13 years of age:	36 (57.1%)	628 (40.0%)	0.057
	Ended from 13 to 16 years:	11 (17.5%)	375 (23.9%)	
	Ended from 16 to 18 or vocational training	9 (14.3%)	351 (22.4%)	
	Completed university studies	7 (11.1%)	215 (13.7%)	
Employment status	Working	10 (11.6%)	325 (17.0%)	0.34
	Unemployed	3 (3.5%)	103 (5.4%)	
	Retired or disable	64 (74.4%)	1,346 (70.4%)	
	Housework or studying	9 (10.5%)	138 (7.2%)	
Monthly income	1650 euros or less	17 (20.7%)	468 (25.7%)	0.670
	From 1650 to 2300 euros	17 (20.7%)	305 (16.8%)	
	From 2300 to 3800	26 (31.7%)	551 (30.3%)	
	From 3800 euros onwards	22 (26.8%)	496 (27.3%)	
Living with a partner	Yes	36 (42.4%)	884 (46.6%)	0.450
Oslo Social Support Scale	Poor	10 (13.2%)	111 (5.9%)	0.004
	Moderate	34 (44.7%)	673 (35.9%)	
	Strong	32 (42.1%)	1,090 (58.2%)	
Self-rated health	Very good, good	14 (16.3%)	754 (39.4%)	<0.001
Cardiovascular disease	Yes	30 (34.9%)	519 (27.1%)	0.110
Musculoskeletal disorders	Yes	58 (67.4%)	1,124 (58.7%)	0.087
Respiratory disease	Yes	12 (14.0%)	254 (13.3%)	0.850
Gastrointestinal disorders	Yes	27 (31.4%)	452 (23.6%)	0.098
Neurological disorders	Yes	29 (33.7%)	250 (13.1%)	<0.001
Cancer	Yes	9 (10.5%)	191 (10.0%)	0.880
Hypertension	Yes	58 (67.4%)	1,228 (64.1%)	0.530
Tobacco use	Currently	5 (6.0%)	240 (12.5%)	0.140
	Former smoker	24 (28.9%)	592 (30.9%)	
	Never smoked	54 (65.1%)	1,081 (56.5%)	
Alcohol use	Daily or weekly	17 (21.0%)	464 (24.4%)	0.330
	Few times a year	14 (17.3%)	422 (22.2%)	
	Not last year or ever	50 (61.7%)	1,018 (53.5%)	
Physically active	Yes	4 (4.9%)	213 (11.2%)	0.072
Weight	Normal	33 (48.5%)	476 (26.3%)	<0.001
	Overweight	16 (23.5%)	795 (43.9%)	
	Obesity	19 (27.9%)	538 (29.7%)	
WHO-5 <50	Yes	8 (30.8)	425 (22.2%)	0.29
PHQ-8 ≥10	Yes	16(48.5)	784 (40.9)	0.38
Consumption of psychiatric medications	Yes	19 (40.4)	565 (29.5)	0.11

Participants were excluded because of missing information on one or more of the primary mental health outcomes (WHO-5, PHQ-8, or prescribed psychiatric medication use). Comparisons were performed before the matching procedure. P values were obtained using Student's *t*-test for continuous variables and the χ^2 test for categorical variables.

Supplementary Figure S1. Factors associated with low emotional well-being in men with diabetes

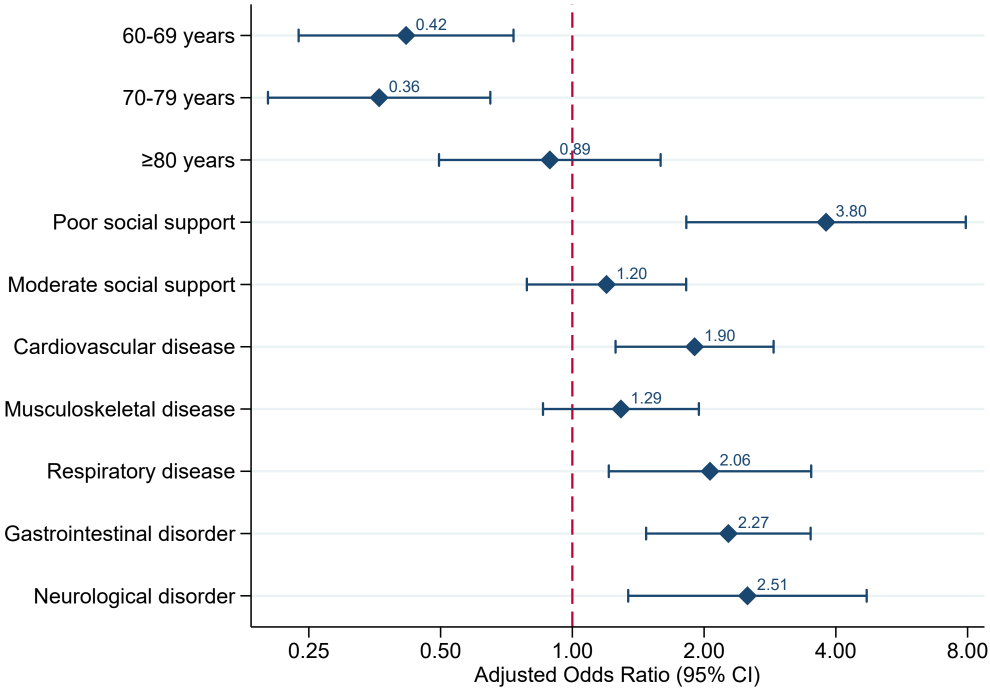

Supplementary Figure S2. Factors associated with depressive symptoms in men with diabetes

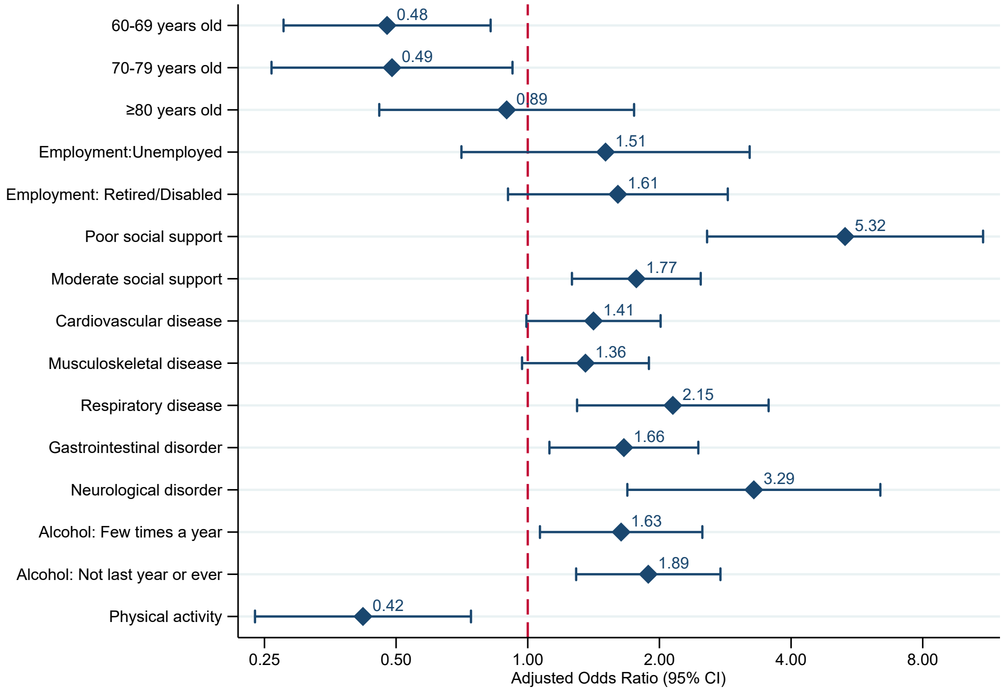

Supplementary Figure S3. Factors associated with psychiatric medication use in men with diabetes

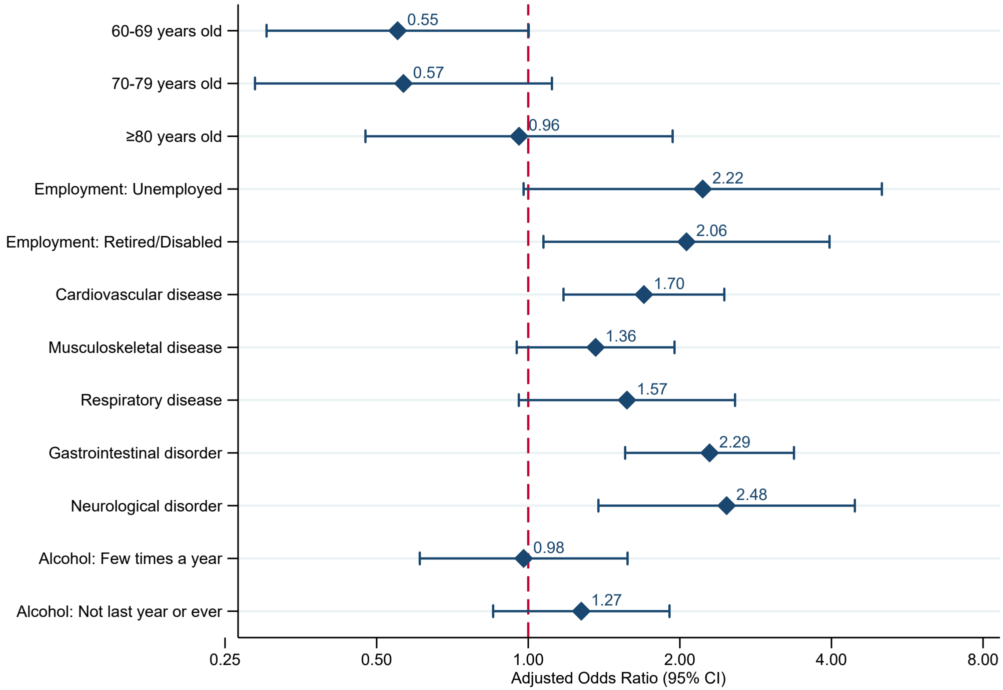

Supplementary Figure S4. Factors associated with low emotional well-being in women with diabetes

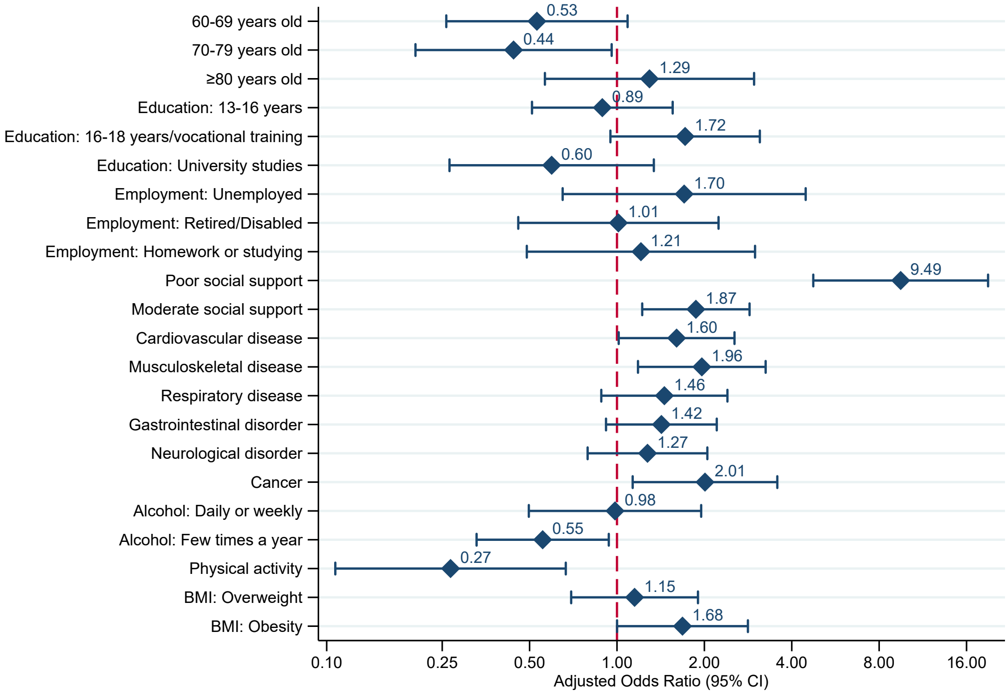

Supplementary Figure S5. Factors associated with depressive symptoms in women with diabetes

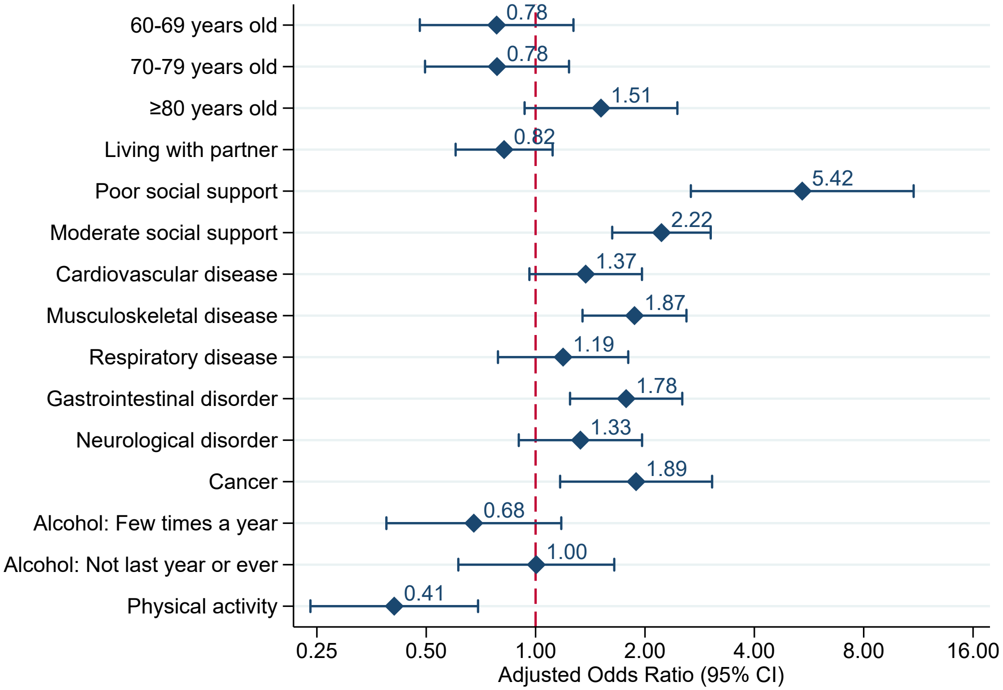

Supplementary Figure S6. Factors associated with psychiatric medication use in women with diabetes

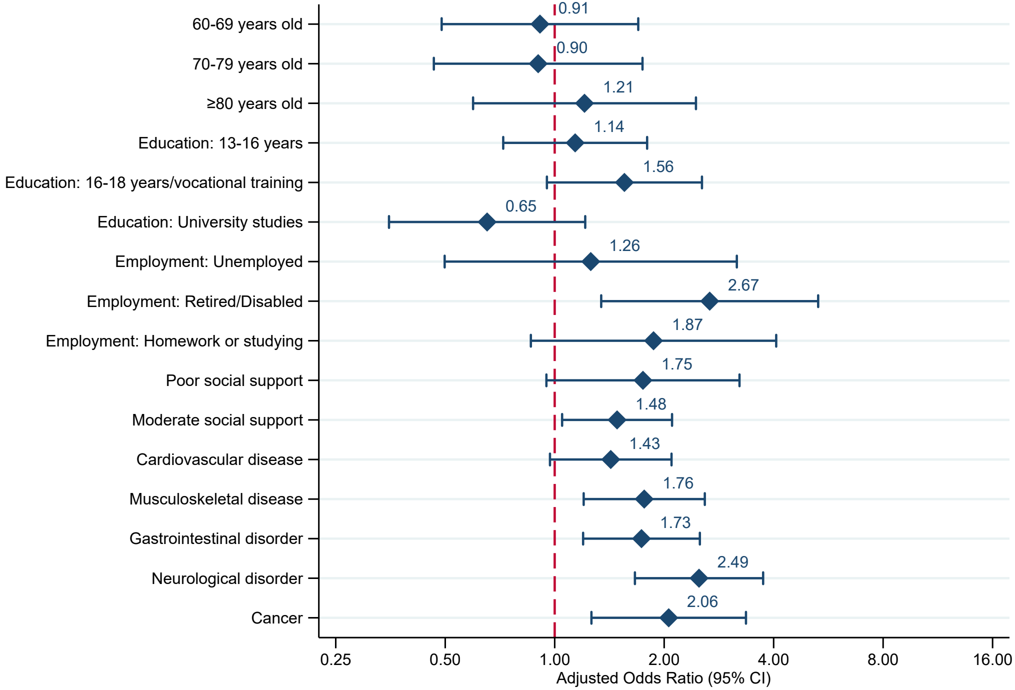

Supplement: Supplementary file 1 [file healthcare-14-02691-s001.zip › healthcare-4418305-supplementary.pdf]
